# Single-cell RNA-seq analyses inform necroptosis-associated myeloid lineages influence the immune landscape of pancreas cancer

**DOI:** 10.3389/fimmu.2023.1263633

**Published:** 2023-12-12

**Authors:** Weiwei Dong, Huixia Zhao, Shanshan Xiao, Liuqing Zheng, Tongqiang Fan, Li Wang, He Zhang, Yanyan Hu, Jingwen Yang, Tao Wang, Wenhua Xiao

**Affiliations:** ^1^ Senior Dept of Oncology, The Fifth Medical Center of People's Liberation Army (PLA) General Hospital, Beijing, China; ^2^ Dept of Oncology, The Forth Medical Center of People's Liberation Army (PLA) General Hospital, Beijing, China; ^3^ Department of Research and Development (R&D), Hangzhou Repugene Technology Co., Ltd., Hangzhou, China

**Keywords:** scRNA-seq, PDAC, TIMs, necroptosis, TME

## Abstract

**Introduction:**

Tumor-infiltrating myeloid cells (TIMs) are key regulators in tumor progression, but the similarity and distinction of their fundamental properties in pancreatic ductal adenocarcinoma (PDAC) remain elusive.

**Method:**

In this study, we conducted scRNA-seq data analysis of cells from 12 primary tumor (PT) tissues, 4 metastatic (Met) tumor tissues, 3 adjacent normal pancreas tissues (Para), and PBMC samples across 16 PDAC patients, and revealed a heterogeneous TIMs environment in PDAC.

**Result:**

Systematic comparisons between tumor and non-tumor samples of myeloid lineages identified 10 necroptosis-associated genes upregulated in PDAC tumors compared to 5 upregulated in paratumor or healthy peripheral blood. A novel RTM (resident tissue macrophages), GLUL-SQSTM1- RTM, was found to act as a positive regulator of immunity. Additionally, HSP90AA1+HSP90AB1+ mast cells exhibited pro-immune characteristics, and JAK3+TLR4+ CD16 monocytes were found to be anti-immune. The findings were validated through clinical outcomes and cytokines analyses. Lastly, intercellular network reconstruction supported the associations between the identified novel clusters, cancer cells, and immune cell populations.

**Conclusion:**

Our analysis comprehensively characterized major myeloid cell lineages and identified three subsets of myeloid-derived cells associated with necroptosis. These findings not only provide a valuable resource for understanding the multi-dimensional characterization of the tumor microenvironment in PDAC but also offer valuable mechanistic insights that can guide the design of effective immuno-oncology treatment strategies.

## Highlights

• ScRNA-seq revealed a heterogeneous tumor-infiltrating myeloid (TIMs) environment in PDAC.
• 10 necroptosis-associated genes were found to be upregulated in PDAC tumors, while 5 genes were upregulated in paratumor or healthy peripheral blood.
• A novel RTM subset, *GLUL* ^-^ *SQSTM1* ^-^ RTM, was found to act as a positive regulator of immunity.
• *HSP90AA1* ^+^ *HSP90AB1* ^+^ mast cells were identified to be pro-immune, while *HSP90AA1* ^-^ *HSP90AB* ^-^ mast cells were anti-immune.
• *JAK3* ^+^ *TLR4* ^+^ CD16 monocytes were found to have anti-immune characteristics, while *JAK3* ^-^ *TLR4* ^-^ CD16 monocytes displayed pro-immune properties.

## Introduction

Pancreatic cancer is a highly malignant tumor of the digestive system, with the most common subtype being pancreatic duct adenocarcinoma (PDAC), and its morbidity and mortality are increasing year by year worldwide (1). The insidious primary lesion determines that more than 80% of cases cannot undergo surgical resection due to regional or distant metastasis, and the postoperative recurrence rate is as high as 85% for resectable patients (2). For radiotherapy and chemotherapy, the mainstay therapeutic strategy, resistances are usually inevitable, leading to limited clinical benefits, especially for advanced patients. Recently, researchers reported the therapeutic potential of immunotherapy by recruiting and activating the host’s T cells to recognize tumor-specific antigens, however, cancer cells developed mechanisms to escape the cytotoxicity effect of T cells. Moreover, the unique immunosuppressive microenvironment of pancreatic cancer hinders the promotion of anti-tumor immune responses through immune checkpoint manipulation (3–5). Therefore, it is urgent to explore novel therapeutic strategies that can significantly improve patient survival and prognosis (6).

Investigations of converting immune “cold” tumors into “hot” tumors are ongoing in immunotherapy. Regulated cell death (RCD), one of the hallmarks of cancer, has been identified as a potential therapeutic target due to its association with anti-tumor immunity. Necroptosis, a recently proposed form of cell death proposed (7), can be inhibited by Necrostain-1 and mediated by receptor-interacting serine/threonine protein kinase 1 (RIPK1) (8). Necroptosis often triggers a robust inflammatory response by releasing cellular contents into the extracellular environment, and this process has been implicated in the pathogenesis and progression of various diseases (9). It is reported that the impact of necroptosis on cancer development, whether inhibitory or promotive, often varies depending on the specific tumor type and stage (10).

The involvement of necroptosis in dysregulated tumor immune microenvironment (TIME) has been demonstrated, especially for myeloid lineages. For instance, damage-associated molecular patterns (DAMPs) were released by tumor cells through necroptosis to stimulate the antigen presentation by dendritic cells (DCs), further enhancing the cytotoxicity of CD8^+^ T cells (11, 12). RIPK3, the effector of necroptosis, contributes to NF-κB activation, tissue repair of DC cells, and infiltration of CD8^+^ T cell (13, 14). Previous studies have reported that the anti-tumor immune response can be activated by NF-κB signaling via necroptosis of fibroblasts (15, 16). However, the regulatory mechanisms of necroptosis in tumor progression in other studies seem to differ from the aforementioned processes. RIPK1 was found to be upregulated in tumor-associated macrophages (TAMs) during M2 Macrophages polarization in a PDAC mice model (17). Necroptosis mediated by RIPK3 promoted the accumulation of immunosuppressive myeloid-derived suppressor cells (MDSCs) in tumor microenvironment (TME) of pancreatic cancer through producing C-x-c motif chemokine ligand 1 (CXCL1) and CXCL5 (18, 19). In an intestinal tumor model, RIPK3 in intermediate MDSC subpopulation was found to increase tumor size (20). Taken together, these findings suggest that myeloid clusters and associated necroptosis may play critical roles in tumor progression and immune evasion.

The exploration of necroptosis-associated myeloid subpopulation can provide a better understanding of the mechanisms underlying immune evasion and therapy resistance in PDAC. Recently, single-cell transcriptomic has made remarkable breakthroughs in deciphering the heterogeneity at the individual cell level. Accumulating evidence has demonstrated the abundance of myeloid cells in tumor immune microenvironment (TIME) of PDAC, serving as key regulators in immune response and treatment resistance (21). By refining the clustering of tumor-associated macrophages (TAMs) in human and mouse samples, researchers have identified significant upregulation of proliferating tissue-resident macrophages and inflammatory macrophages in PDAC TIME received chemotherapy. Conversely, monocyte-derived antigen-presenting cells (APCs) and Marco+ macrophages highly expressed the scavenger receptor MARCO, showed decreased expression. Results from multiplex immunohistochemistry (mIHC) further supported the chemotherapy resistance of proliferating tissue-resident macrophages (22). The deficiency of DCs has been linked to dysfunctional T cell-mediated immunity in early-stage PDAC, indicating their vital role in immune escape and tumor progression (23). Although some progress have been made, further detailed characterization of myeloid cell lineage is needed, and the therapeutic application of myeloid cells in pancreatic cancer remains limited.

This study aims to further elucidate the unique microenvironment of PDAC, explore its intrinsic mechanisms in the tumor occurrence and progression, and provide a potential novel approach for the treatment of PDAC patients. Leveraging a publicly available scRNA-seq resource (24), we revealed a tumor-associated myeloid environment in PDAC. Specifically, we identified upregulated necroptosis genes and immune-related novel clusters in PDAC. Furthermore, we discovered cell-specific signaling pathways and receptor-ligand pairs within these new clusters, which have the potentially to either promote or suppress tumor development. In general, utilizing this unique resource, we analyzed myeloid cell lineages, necroptosis-associated networks, and cell-cell crosstalk in PDAC. This sheds light on the myeloid ecosystems underlying PDAC initiation and progression, and may provide a myeloid-modulating therapeutic strategies from pre-clinical models to pancreatic cancer treatment.

## Methods

### Data source and preprocessing

**Figure 1:**
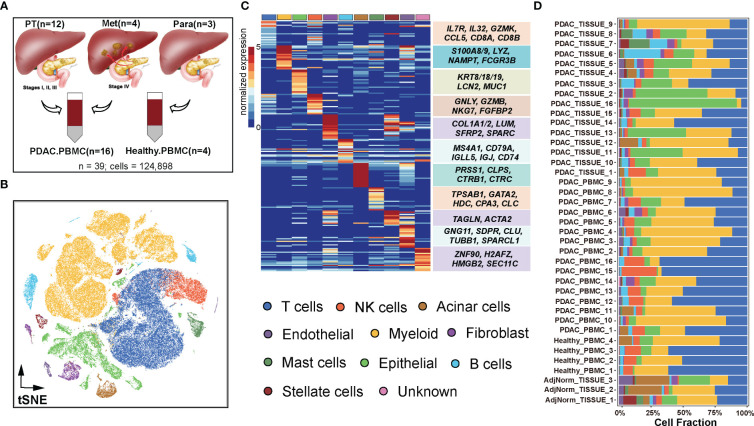
Dissection of the tumor microenvironment in PDAC. **(A)** Samples were collected from GSE155698. **(B)** Visualization of single-cell RNA-seq data of 124,575 cells by t-SNE. **(C)** Single-cell resolution heatmap of top expressed genes for each cell type. **(D)** Proportions of 11 cell types among 39 samples including 16 primary or metastatic tumor tissues coupled with 16 PDAC PBMC samples, 3 paratumor tissues and 4 healthy PBMC samples.

The PDAC dataset GSE155698 (24) was downloaded from the GEO database, including 12 primary tumor (PT) patients, 4 metastatic (Met) patients, 3 adjacent normal pancreas tissue (Para) patients, and all samples were coupled with peripheral blood (**Figure 1A**). The original dataset contained a total of 25,236 genes and 142,353 cells. The raw UMI count matrices were processed using the R package Seurat (version 3.2.3) (25). The data underwent several filtering steps: 1) cells with a low number of unique detected genes (< 200) and a high number of 5000 were removed; 2) cells with more than 30,000 UMIs were discarded; 3) cells with mitochondrial content higher than 30% were removed; 4) cell cycle genes were regressed out. After excluding low-quality cells, 124,575 single cells remained for downstream analysis. Additionally, another publicly available scRNA-seq data from CRA001160 (26), including a total of 57,539 cells from 24 primary PDAC tumors and 11 control pancreases, was utilized to validate the findings.

Public clinical data and gene expression information were retrieved from the TCGA database (https://www.cbioportal.org/). A total of 178 samples from the TCGA-PAAD (Pancreatic adenocarcinoma) cohorts were included for further analysis.

### Sing-cell RNA-seq data clustering and dimensional reduction

First, we performed data normalization on the merged data using the *NormalizeData* function and identified the first 2000 highly variable genes through the *FindVariableFeatures* function, which is based on the variance stabilization transformation (“*vst*”). Simultaneously, all genes were scaled using the *ScaleData* function, and the *RunPCA* function was applied to reduce the dimensionality of the data using PCA for previously identified highly variable genes. We selected a dimensionality reduction of 30 (dim = 30) and clustered the cells using the *FindNeighbors* and *FindClusters* functions with a resolution of 1.2, enabling the identification of distinct cell clusters. To further reduce dimensionality and visualize the data, we employed the UMAP and tSNE methods using the top 30 principal components. Specifically focusing on myeloid cell types, we repeated the clustering protocol to identify clusters within the aforementioned myeloid lineages. To address batch effects, we utilized the *runHarmony* function from the Harmony package (version 0.1.0) (27). Finally, we employed the *FindAllMarkers* function to screen the marker genes of 40 subgroups, considering a log-fold change (logfc) threshold of 0.25 for differential expression and a minimum percentage (min.pct) of 0.25 for the expression ratio of the least differentially expressed genes. We applied a corrected p threshold of less than 0.05 to screen the significant marker genes.

### Cell type identification

The annotation of each cell cluster was confirmed by the expression of canonical marker genes. Epithelial cells were identified using the higher expression of *EPCAM*, *ACTA2*, *KRT7*, *KRT8*, *KRT18*, *KRT19*, *CDH1*, *PRSS1*, *CTRB2*, *REG1A*, *CLU*, *MKI67*, *SPINK1*, *TFF1*, and *MUC1*, and other cell types were annotated using: T cells (*CD3D*, *CD3E*, *CD3G*, *CD4*, *CD8A*, *IL7R*, and *LEF1*), B cells (*MS4A1*, *CD79A*, *CD79B*, *CD52*, *CD19*, *SDC1*, *IGJ*, *IGLL5*, *CXCR4*, *KIT*, *CD27*, and *HLA-DRA*), NK (natural killer) cells (*NCR3*, *FCGR3A*, *NCAM1*, *KLRF1*, *KLRC1*, and *CD38*), acinar cells (*PRSS1*, *CTRB1*, *CTRB2*, *REG1B*, *SPINK1*, and *AMY2A*), mast cells (*TPSAB1*, and *CPA3*), fibroblast (*LUM*, *DCN*, *COL1A1*, *ACTA2*, *SPARC*, *CDH11*, *PDGFRA*, *PDGFRB*, *COL3A1*, *RGS5*, *IGFBP7*, *PDPN*, *MCAM*, *IL6*, *APOE*, *GLI1*, *GLI2*, *GLI3*, and *PDGFA*), myeloid cells (*CD14*, *ITGAM*, *MNDA*, *MPEG1*, *ITGAX*, *FCGR3A*, *FCGR3B*, *APOE*, *C1QA*, *MARCO*, *LYZ*, and *HLA-DRA*), stellate cells (*RGS5*, *ACTA2*, *PDGFRB*, and *ADIRF*), and endothelial cells (*CDH5*, *PLVAP*, *VMF*, *VLDN5*, *KDR*, and *PECAM1*).

Among myeloid cells, cell clusters were identified using genes previously reported. Mast cells were identified by the high expression of *KIT*, *CPA3*, and *TPSAB1*, and other myeloid lineages were annotated using: granulocytes (*CXCR2*, *FCGR3B*, *IFTIM2*, *SLC25A37*, *IL1R2*, *CXCR1*, *SIRPA*, and *S100A8*), macrophages (*C1QC*, *C1QA*, *APOE*, *CCL4*, *PLTP*, and *IL1B*), CD14 monocyte (*CD14*, *FCN1*, *S100A8*, and *S100A9*), CD16 monocyte (*FCGR3A*, *LST1*, and *LILRB2*), and DCs (dendritic cells) (*IL7R*, *CCR7*, *GZMB*, *LYZ*, *IL3RA*, and *IL32*).

### Tissue distribution of clusters

We quantified the tissue preference of each cluster by calculating the ratio of observed to expected cell numbers (*Ro/e*) in different tissue (28, 29). The expected cell numbers for each combination of cell clusters and tissues were determined using the chi-square test. A cluster was considered enriched in a specific tissue if *Ro/e* value was greater than 1.

### Differential expression and pathway analysis

To identify differentially expressed genes between two groups of clusters, we used the Wilcox method in the *FindMarkers* function in *Seurat* to evaluate the significance of each gene, with multiple hypothesis correction using the *Benjamini-Hochberg* procedure. Genes with adjusted *P*-values less than 0.05 were considered as differentially expressed genes. In addition, the log2 fold change (log2FC) for each gene was calculated by subtracting the log2 transformed mean count in each group.

KEGG (Kyoto Encyclopedia of Genes and Genomes) pathway enrichment was performed using the *clusterProfiler* package (version 3.14.3) (30), with a *Benjamini-Hochberg multiple testing adjustment*. Gene sets with FDR-corrected P < 0.01 were considered to be significantly enriched.

### TCGA data analysis

Two endpoints (overall survival (OS) and disease-free interval (DFI) from the TCGA-PAAD) were used to analyze patients’ clinical outcomes. We employed the Cox proportional hazards model implemented in the *survival* package to assess the correlation between selected genes and patients’ survival. Kaplan-Meier survival curves were generated using the R function *ggsurvplot* from the *survminer* package.

Specifically, to examine the relationship between clusters and patients’ survival, we utilized their signature genes. The signature genes including the following categories: macrophage (*C1QC*, *C1QA*, *APOE*, *MACRO*, *INHBA*, *IL1RN*, *CCL4*, *NLRP3*, *EREG*, *IL1B*, *LYVE1*, *PLTP*, *SEPP1*), granulocyte (*FCGR3B*, *IFITM2*, *CXCR2*, *S100A8*, *SLC25A37*, *CXCR1*, *IL1R2*), CD14 monocyte (*CD14*, *FCN1*, *S100A8*, *S100A9*, *S100A12*, *VCAN*, *CD36*), CD16 monocyte (*FCGR3A*, *LST1*, *LILRB2*, *IFITM2*, *SIGLEC10*, *CX3CR1*, *LILRB1*, *LIBRA1*, *TCF7L2*, *MTSS1*, *RHOC*), DCs (*GZMB*, *JCHAIN*, *MZB1*, *CLIC3*, *CXCL8*, *IL7R*, *CCR7*, *MMP7*, and *IL32*), and mast cells (*KIT*, *CPA3*, *TPSAB1*, *HDC*, *GATA2*, *HPGDS*, *TPSD1*, *SLC18A2*, *MS4A2*, *IL1RL1*, and *VWA5A*). The mean expression of the signature genes was used to classify samples into a high and low groups based on risk score (high: risk score > 0; low: risk score ≤ 0). The Cox model was employed to adjust for OS and status in the survival analysis.

### Cell-cell interaction analysis

To investigate the potential interactions between different cell types in the TME of PDAC, we conducted cell-cell interaction analysis using *CellChat* (v1.1.3), which integrates a curated repository of ligand-receptor (L-R) pairs and employs a statistical framework (31). We combined CD4^+^T cells, CD8^+^T cells, B cells, NK cells, and epithelial cells with the newly identified clusters, including *GLUL* ^-^ *SQSTM1* ^-^ RTM, *GLUL* ^+^ *SQSTM1* ^+^ Macro., *HSP90AA1* ^+^ *HSP90AB1* ^+^ Mast and *JAK3* ^+^ *TLR4* ^+^ Mono. Interactions networks between cell clusters were investigated.

### RNA fluorescence *in situ* hybridization

The samples used for RNA FISH were obtained from tumor paraffin sections of patients diagnosed with PDAC. Isolated cancer-associated cells were adhered onto laminin coated #1 coverslips (ThermoScientific) were fixed for 10 min at room temperature with Fixation Buffer (3.7% formaldehyde in PBS), washed twice in 1x PBS and permeabilized with 70% EtOH at 4°C for at least an hour. RNA FISH was performed using 20-mer Stellaris Biosearch Probes for LINCMs and core gene conjugated to Quasar 670 or CAL Fluor Red 610. Briefly, cells were washed with Wash Buffer (10% formamide in 2x SSC) prior to overnight 37°C hybridization with target probes (125 nM) in Hybridization buffer (100 mg/ml Dextran Sulfate, 10% Formamide in 2x SSC). After hybridization, cells were washed in Wash Buffer for 30 min at 37°C, counterstained with DAPI (5 ng/ml in Wash Buffer) for 30 min at 37°C, and washed in 2x SSC at room temperature. Coverslips were transferred onto glass slides with mounting medium (Vectashield) and imaging was performed immediately on upright microscope (Nikon, Ni-E) with 100x Objective (Nikon) on a cooled CCD/CMOS camera (Qi-1, Qi-2, Nikon).

For the notable exception of S100A4, SQSTM1 and GLUL RNA FISH co-staining, RNA FISH was performed using 50-mer ZZ ACD RNAScope probes due to the short unique sequence of the antibodies available for probe design. Cells were fixed and permeabilized as described above in 70% EtOH, washed in 1x PBS and 1x Hybwash buffer for 10 and 30 min, respectively. They were then incubated with 1x Target Probe Mix at 40°C for 3 hours. Cells were washed thrice in 1x Hybwash at room temperature, incubated in 1x Pre Amp Mix for 40 min at 40°C, washed thrice in 1x Hybwash at r.t.p, incubated in 1x Amp Mix for 30 min at 40°C, washed twice in 1x Hybwash before incubation in 1x Label Probe Mix (Alexa Fluo 488, ATTO0550) at 40°C for 25 min. Cells were washed thrice in 1x Hybwash in dark at r.t.p, counterstained with DAPI (5ng/ml) prior to mount and imaging.

### Statistical analysis

All statistical analyses were conducted using R software. Comparisons between two groups of samples were evaluated using Wilcoxon rank-sum test (Mann-Whitney U-test) for statistical analysis. Statistical significance was denoted as *P < 0.05, **P < 0.01, ***P < 0.001.

## Results

### Overall characteristics of the cell cluster composition in pancreatic cancer

To gain a comprehensive understanding of TME, and explore its heterogeneity between PDAC and normal tissues, we investigated 39 PDAC samples consisting of primary tumors, metastatic tumors, adjacent normal tissues and paired peripheral blood (**Figure 1A**) from GSE155698 (24). Based on canonical cell markers mentioned in Methods (**Table S1**), a total of 124,575 cells were classified into distinct cell populations, including T cells (39,372 cells, 31.61%), myeloid cells (48,054, 38.57%), epithelial cells (14,998, 12.04%), NK cells (7,758, 6.23%), fibroblast (2,617, 2.10%), B cells (4,548, 3.65%), acinar cells (2,488, 2.00%), mast cells (2,086, 1.67%), stellate cells (1,324, 1.06%), endothelial cells (1,238, 0.99%) and minor unknown cells (92, 0.74%) (**Figures 1B, C**, **S1A**).

Compared to primary tumors and paracancerous samples, metastatic tumors exhibited higher composition of T cells (34.52%, 27.55%, 23.59%, respectively) and epithelial cells (15.17%, 12.54%, 9.47%) (**Figure S1B**, **Table S2**). Conversely, compared to primary and metastatic tumor samples, adjacent normal tissues had higher proportions of stromal cells, including acinar cells (14.27%, 1.11%, 1.21, respectively), stellate cells (6.26%, 0.95%, 0.57%) and endothelial cells (3.90%, 0.92%, 0.89%) (**Figure S1B**, **Table S2**). As for PDAC samples across clinical stage I to IV, the proportions of epithelial cells continued to rise, from 5.58% to 15.17%, while myeloid cell compositions decreased from 71.63% to 35.13% (**Figure S1C**, **Table S2**). Among the PDAC samples, T cells (28.68%), myeloid cells (40.25%), and epithelial cells (12.86%) were the most abundant populations. In normal samples, T cells (51.88%), myeloid cells (27.00%), and NK cells (9.26%) accounted for 88.14% of the cell population (**Table S2**, **Figure S1D**). The peripheral blood from PDAC patients had a higher proportion of T cells (36.87% vs. 22.41%), myeloid cells (41.46% vs. 33.53%), and NK cells (7.53% vs. 3.95%) compared to solid tissue, whereas the proportion of epithelial cell was higher in solid tissue (20.19%) than peripheral blood (7.37%) (**Figure S1E**, **Table S2**).

Similar to previous studies (26, 32), there was significant variation in the portions of epithelial, stromal, and immune cells among the samples, which could be attributed to intrinsic differences in tumor stages or specific locations within tumor where biopsies were taken (**Figure 1D**). For example, PDAC patients 15 and 16 (stage II) exhibited a highly immune-rich microenvironment, with nearly 70% T cells in peripheral blood compared to only 10% in solid tissue. Furthermore, patients 15 and 16 (stage II) had higher T cell portions (70%) than patients 2 and 3 (stage IV, approximately 20%). These findings indicated that the formation and progression of metastases in PDAC may necessitate a more immunosuppressive TME compared to primary tumors.

### B cell may play a tumor-suppressive role in PDAC

Subsequently, to assess the clinical significance of these cell types in PDAC, we identified the top 20 genes that predominantly determined the identity of each cell type through ROC analysis. The correlation between the expression levels of these genes and the patient prognosis was then computed using multivariate Cox regression on TCGA-PAAD data (**Table S3**). Our analysis revealed that genes exclusively expressed in C3 (epithelial cell) (ave.cox = 0.182), C13 (epithelial cell) (ave.cox = 0.182), C21 (epithelial cell) (ave.cor = 0.193) were associated with poor prognosis (**Figure S2A**), where ave.cox represents the average Pearson correlation coefficient. On the other hand, genes expressed in C0 (T cells) (ave.cox = -0.076) and C14 (B cells) (ave.cox = -0.062) were correlated with a favorable prognosis in PDAC, suggesting potential tumor-suppressive functions of these cells. It is worth noting that B cells are prominent features of PDAC tumors, although their roles in this disease remain controversial (33). Notably, higher expression levels of genes exclusively expressed in the C14 and C38 B cell types (such as *BCL11A* and *DNASE1L3*) were positively associated with favorable prognoses(**Figure S2B**, **Table S3**), indicating the tumor-suppressive functions of C14 and C38 cells in the PDAC microenvironment. Correlation analysis revealed that *BCL11A* (*R* = 0.419, *p* = 3.52e-09) and *DNASE1L3* (*R* = 0.689, *p* < 2.2e-16) were positively correlated with CD8A (**Figure S2C**). Clinical outcomes demonstrated that higher expressions of *BCLAA1* and *DNASE1L3* were significantly associated with improved survival (**Figures S2E, F**). Additionally, these genes exhibited higher expression values in PDAC tumors compared to normal samples (**Figure S2D**), suggesting B cells may exert tumor-suppressive roles as tumor-infiltrating B cells. Consistent with our findings, previous studies have demonstrated that tumor-infiltrating B cells are a positive prognosis factor, both in PDAC and other cancers (34, 35).

### Myeloid cells exert immune-suppressive potential

**Figure 2:**
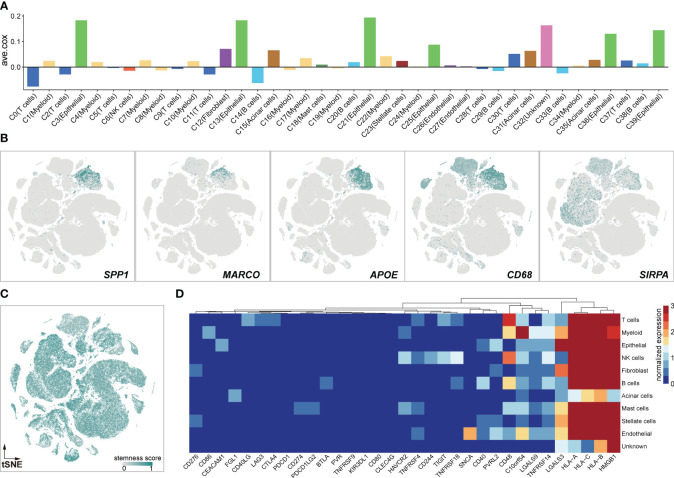
Myeloid cells exert immune-suppressive potentials. **(A)** Bar Graphs illustrating the average coefficients (ave.cor) for the prognostic effect of genes exclusively expressed in each clusters. Positive values of ave.cor indicate the associations with anti-tumor immunity and good clinical outcomes, opposite from negative ave.cor values. **(B)** Expression landscapes of immunosuppressive markers of *SPP1, MARCO, APOE, CD58*, and *SIRPA*. **(C)** The high stemness score profile of myeloid cells calculated by *scCancer* (36). **(D)** Heatmap of immune checkpoint inhibitors in 11 cell clusters.

The presence and functional activities of myeloid cells in tumors have garnered increasing interest due to their relevance as modulators of anticancer therapies and potential targets for specific treatment. In this study, we focused on unraveling the potential roles of myeloid cells in PDAC (**Figure 2**). Correlation analysis showed that the genes exclusively expressed in C1 (ave.cox = 0.023), C4 (ave.cox = 0.019), C7 (ave.cox = 0.026), C10 (ave.cox = 0,023), C17 (ave.cox = 0.034), C22 (ave.cox = 0.042) (myeloid cell) were associated with poor prognosis (**Figure 2A**), suggesting the immune-suppressive functions of myeloid cells in the microenvironment of PDAC. Besides, Immune-suppressive markers as previously reported (37), *SPP1*, *MACRO*, *APOE*, *CD68*, and *SIPRA*, were exclusively expressed in myeloid cells (**Figure 2B**). Additionally, myeloid cells had a relatively higher stemness score compared to other stromal cells (**Figure 2C**).Previous studies have demonstrated that cancer progression involves a gradual loss of differentiated phenotype and the acquisition of progenitor-like, stem cell-like features (38). Furthermore, myeloid cells exhibited heterogeneous expression of immune checkpoint receptors (*CD86*, *HAVCR2*, *CD48*, and *VSIR*) (**Figure 2D**). Collectively, these findings suggest that myeloid cells may play an immune-suppressive role in the PDAC tumor environment, consistent with previous observations and supporting the notion that myeloid cells are a key immunosuppressive component in TME (39).

### Characterization of major myeloid cell lineages

**Figure 3:**
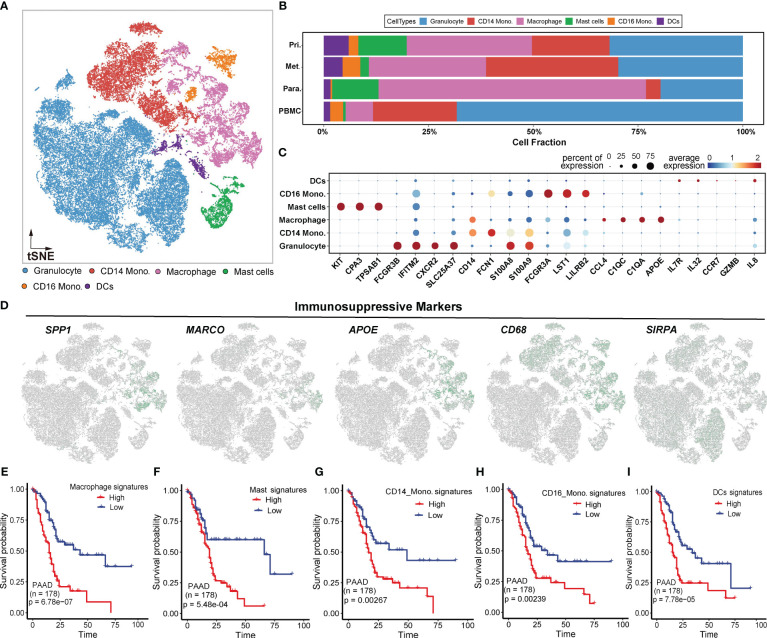
Myeloid-derived cell components in PDAC. **(A)** t-SNE plot showing 6 myeloid clusters of samples from PDAC patients. **(B)** Proportion of each myeloid cell lineage from the primary tumor, metastatic tumor, paracancerous tissue, and peripheral blood. **(C)** Bubble plot showing selected cell type-specific markers across all clusters. The size of dots represents the fraction of cells expressing a particular marker, and the intensity of the color indicates the levels of average mean expression. **(D)** t-SNE plots showing the expression of specific immunosuppressive markers, which were denoted in Figure B, in the myeloid subclusters. **(E–I)**. Kaplan-Meier survival analyses of some myeloid subclusters markers, including macrophage, mast cells, CD14 monocyte, CD16 monocyte, and DCs.

To investigate tumor-infiltrating myeloid cells (TIMs) in PDAC, we firstly excluded all cells from healthy samples, resulting in 45,859 myeloid cells for further analysis. Subsequently, we performed unsupervised clustering and cell annotation of myeloid cells using canonical markers (**Figures 3A, C**), as described in the Methods section. It revealed the presence of 6 distinct subclusters within the myeloid lineage, including granulocytes (24,786, 54.0%), CD14 monocytes (8,713, 19.0%), macrophages (7,767, 16.9%), mast cells (1,999, 4.4%), CD16 monocytes (1,270, 2.8%), and dendritic cells (DCs) (1,324, 2.9%) (**Figure 3B**).

To visualize the distribution of cell populations of myeloid subclusters across different histologic types, we utilized unbiased hierarchical clustering algorithms and supervised annotation on PBMC samples(**Figures S3A, B**). Compared to adjacent normal tissues, tumor tissues exhibited overall increases in DCs, CD14 monocytes, and CD16 monocytes, indicating a redirected immune response (**Figure S3C**). Besides, PBMC samples predominantly consisted of granulocytes, while tumor and paratumor tissues exhibited abundant macrophages (**Figure S3C**). The distict cellular compositions suggested a heterogeneous TIMs environment in tumor.

We subsequently investigated the expression patterns of immune-suppressive markers mentioned above. The results revealed elevated expression of these markers in macrophages (**Figure 3D**), suggesting that macrophages may contribute to the immunosuppressive effects of TIMs in PDAC. To further assess the clinical impact of the signature (**Figure 3C**) for myeloid subclusters, including macrophages, mast cells, CD14 monocytes, CD16 monocytes and DCs, we utilized an independent PAAD cohort from TCGA. Patients with high expression of signature genes exhibited worse OS compared to those with low expression(two-sided log-rank test p < 0.01) (**Figures 3E–I**). These findings further underscored the immunosuppressive effects of myeloid lineages at the bulk level.

### PDAC tumor progression is associated with necroptosis

**Figure 4:**
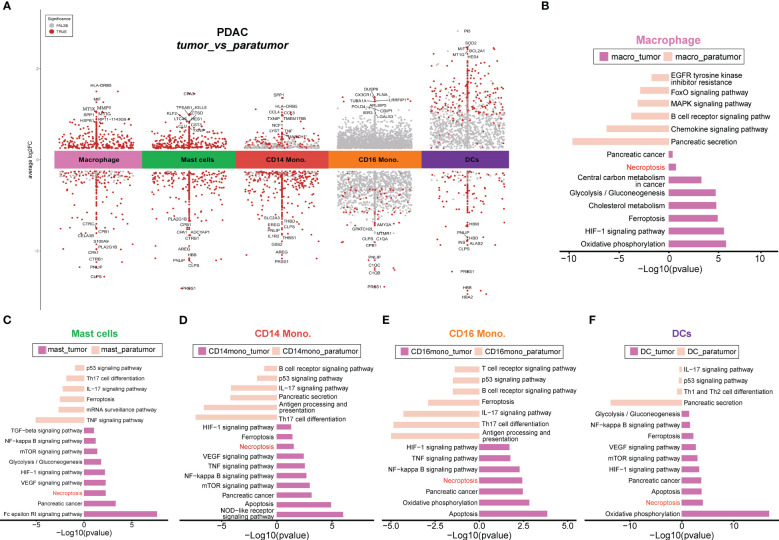
Differential gene analyses of PDAC tumors and paracancerous tissues. **(A)** Differential gene expression analysis showing up- and down-regulated genes across all cell types between tumor and paratumor samples from PDAC patients. The top 10 DE genes were shown, and the points dotted in red indicate significant genes. An adjusted p-value < 0.01 is indicated in red, while an adjusted p-value ≥ 0.01 is indicated in black. **(B–F)**. Differential pathways enriched in tumor and paratumor for each cell type.

In order to comprehensively investigate the diverse range of myeloid cell populations in PDAC, we first performed tissue prevalence analysis. Compared to paratumor samples, macrophages and mast cells were highly enriched in tumor tissues, suggesting the coexistence of host immune response and tumor escape in the PDAC milieu. Moreover, macrophages exhibited a higher enrichment in paratumor samples compared to primary and metastatic tumor samples, in contrast to CD14 monocytes and granulocytes (**Figure S3C**, left). Moreover, macrophages and mast cells exhibited a preferential enrichment in PDAC tissues rather than peripheral blood samples (**Figure S3C**, middle). Subsequently, we performed differential gene expression (DGE) analysis between tumor and paratumor samples (**Figure 4A**) and gene set enrichment analysis of each cluster’s upregulated genes (**Figures 4B-F**). Strikingly, gene ontology (GO) characteristics related to necroptosis were detected across all myeloid lineages in tumor samples. Necroptosis can either elicit robust adaptive immune responses that may impede tumor progression, or it can recruit inflammatory responses that may potentially facilitate tumorigenesis, cancer metastasis and the generation of an immunosuppressive tumor microenvironment (40).

To investigate the potential enrichment of necroptosis in peripheral blood, we only kept myeloid cells from peripheral blood samples (**Figure S3A**). Compared with normal blood samples, blood samples from PDAC patients showed higher proportions of CD14 monocytes and lower proportions of DCs, and CD16 monocytes (**Figure S3B**), indicating a heterogeneous myeloid environment in peripheral blood. However, myeloid lineages showed comparable enrichment in peripheral blood except for DCs, CD16 monocytes, and macrophages (**Figure S3C**, right). We performed DGE and enrichment analyses of PBMC samples from PDAC or healthy individuals, and results demonstrated the presence of necroptosis in PBMC samples from PDAC (**Figures S3D-I**), indicating that the necroptosis event was not tissue-specific. To investigate the differences between tumor and paratumor tissues, we excluded myeloid cells from peripheral blood samples. DGE and enrichment analyses showed that necroptosis remained specifically enriched in all myeloid lineages within tumor tissues (**Figures S4A-F**). Moreover, we removed myeloid cells from adjacent normal samples to compare the differences between tumor tissue and peripheral blood from PDAC. The results showed that necroptosis was specifically enriched in all myeloid lineages from tumor tissues, rather than peripheral blood (**Figures S5A-F**), suggesting a propensity for necroptosis events to occur in solid tumor tissues. Detailed information on all DEGs and necroptosis-associated DEGs could be found in **Tables S4**,**S5**, respectively.

**Figure 5:**
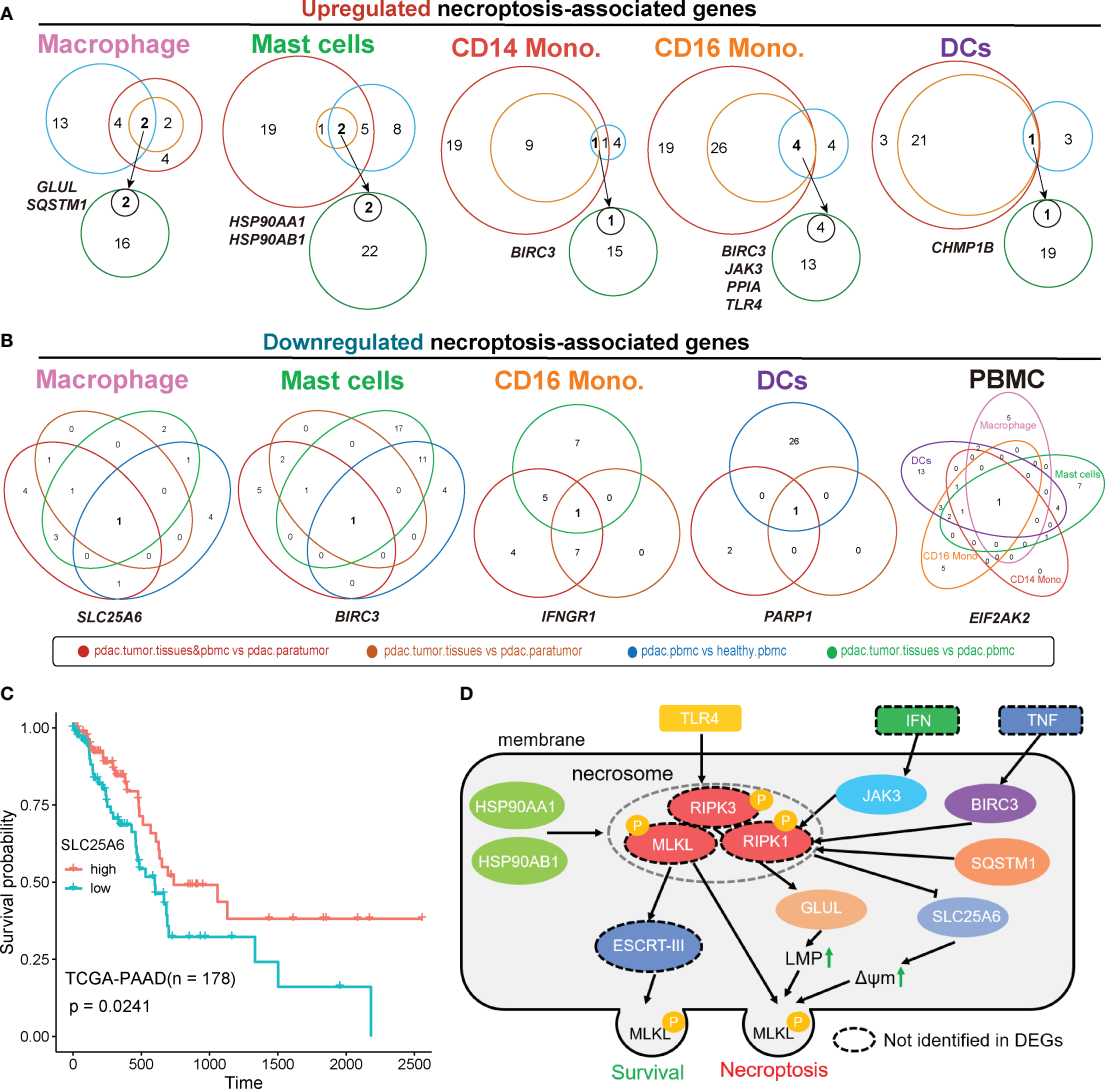
Overlapped differentially expressed genes were associated with necroptosis. Analysis of overlapped necroptosis-associated genes up-regulated **(A)** or down-regulated **(B)** in tumor for each cell type. **(C)** TCGA survival analysis of SLC25A6. **(D)** The predicted regulatory pathways of overlapped necroptosis-associated genes we identified based on public Necroptosis pathway network. Genes in dashed-circles were not identified in overlapped DEGs. ‘pdac.tumor.tissues&pbmc vs pdac.paratumor’, primary tumor tissues, metastatic tumor tissues and PBMCs samples from PDAC patients vs paratumor samples from PDAC patients.; ‘pdac.tumor.tissues vs pdac.paratumor’, primary tumors and metastatic tumors from PDAC patient vs paracarcinoma tissues from PDAC patient; ‘pdac.pbmc vs healthy.pbmc’, PBMCs of PDAC Patients vs PBMCs from healthy controls; ‘pdac.tumor.tissues vs pdac.pbmc’, primary tumor tissues and metastatic tumor tissues of PDAC patients vs PBMCs of PDAC patients; LMP, lysosome membrane permeabilization; ΔΨm, mitochondrial membrane potential.

We next performed overlapping analyses to find key necroptosis-pathway-associated (NPA) DEGs that exhibited significant up- and down-regulation within each myeloid lineage (**Figure 5**; **Figure S6**). The results revealed specific patterns in the expression of NPA genes in different myeloid cell types within tumor tissues. In macrophages from tumor samples, two NPA genes, *GLUL* and *SQSTM1*, were found to be up-regulated (**Figure 5A**), while *SLC25A6* exhibited down-regulation (**Figure 5B**). Besides, in mast cells, two NPA genes *HSP90AA1* and *HSP90AB1* were up-regulated in tumor tissues (**Figure 5A**), whereas *BIRC3* was down-regulated (**Figure 5B**). Interestingly, *BIRC3* displayed an opposite expression pattern, being up-regulated in CD14 monocytes and CD16 monocytes but down-regulated in mast cells (**Figure 5B**), indicating potential distinct roles of this NPA gene in different cell types. Additionally, in CD16 monocytes, the remaining NPA genes, *JAK3*, *PPIA*, and *TLR4* were up-regulated in tumor tissues (**Figure 5A**), while *IFNGR1* was down-regulated in tumor samples (**Figure 5B**). In the case of DCs, the NPA gene, *CHMP1B* was up-regulated in tumor tissues (**Figure 5A**), while *PARP1* was down-regulated in tumor samples (**Figure 5B**).

Based on the Necroptosis pathway network (https://www.kegg.jp/pathway/map04217), several NPA DEGs that identified within PDAC myeloid cells were involved in this network, thereby the interactive relationship was mapped. This approach allowed us to gain insights into the underlying contribution of necroptosis in the context of myeloid cell-mediated immune responses within TME of PDAC. Of note, we designated this mapping as the representation of the necroptosis pathway associated with PDAC myeloid cells, while experimental studies are necessary to confirm their precise roles and interactions(**Figure 5D**). In this predicted simplified model, *TLR4* acts as an upstream regulator that promotes the phosphorylation of *RIPK3*, a key regulator of necroptosis (41). This phosphorylation event leads to the subsequent phosphorylation of *GLUL*, which contributes to increased lysosome membrane permeabilization (LMP), a common phenomenon in cancer cells (42). Besides, *JAK3*, a downstream signaling molecule of IFN, *BIRC3*, a downstream molecule of TNF, and *SQSTM1*, a dissociated molecule, work together to promote the phosphorylation of *RIPK1*, another core regulator of necroptosis (41). The phosphorylated *RIPK1*, in turn, represses the expression of *SLC25A6*, result in a transient increases in mitochondrial transmembrane potential (ΔΨm), which is highly related to cancer malignancy (43). Moreover, *HSP90AA1* and *HSP90AB1* can simultaneously promote the phosphorylation of necrosome, including *RIPK1*, *RIPK3*, and *MLKL*. This leads to the activation of various necroptosis pathways, such as MLP, ΔΨm and mitochondrial fission (44). Mitochondrial fission facilitates the proliferation, metastasis, and drug resistance of cancer cells (45). Despite their necroptosis-promoting function, HSP90AA1 and HSP90AB1 can also act as upstream regulator of ESCRT-III, which helps maintain membrane integrity during the initiation of necroptosis, thereby promoting cell survival (46).

**Figure 6:**
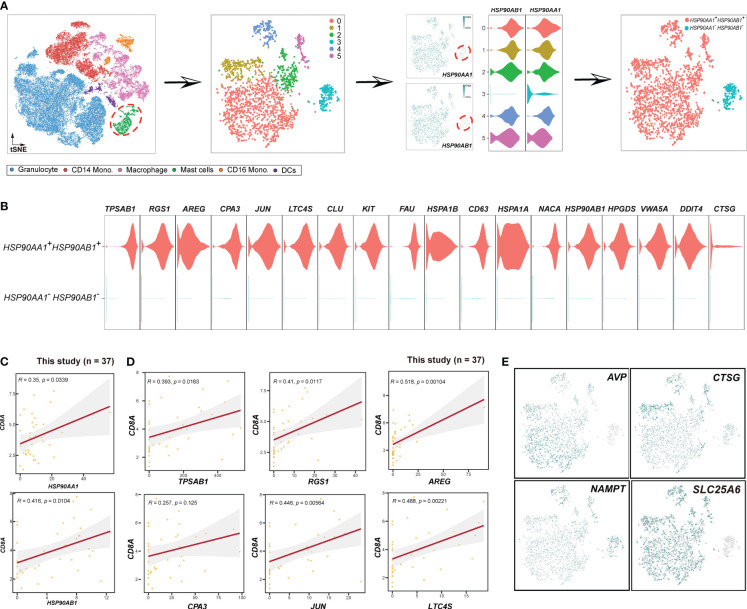
Schema to identify *HSP90AA1* ^+^ *HSP90AB1* ^+^ mast cells. **(A)** Schema showing the procedures to distinguish mast cells based on *HSP90AA1* and *HSP90AB1*. **(B)** Violin plots displaying the expression of *HSP90AA1* ^+^ *HSP90AB1* ^+^ mast cluster-specific genes. **(C)** Scatterplot showing the Spearman correlation between the *HSP90AA1*, *HSP90AB1*, and CD8A in this study. **(D)** Scatterplot illustrating the correlations between the top 6 *HSP90AA1* ^+^ *HSP90AB1* ^+^ mast cluster-specific genes and *CD8A* in this study. **(E)** Expression patterns of cytokines (*AVP*, *CTSG*, *NAMPT*, *SLC25A6*) in mast cells.

To explore the clinical relevance of necroptosis-associated genes that were down-regulated in tumor (**Figure 5B**), we conducted survival analysis. The results revealed that higher expression of *SLC25A6* was correlated with improved survival outcomes (**Figure 5C**). Interestingly, we observed a significantly higher expression of *SLC25A6* in *HSP90AA1* ^+^ *HSP90AB1* ^+^ mast cells compared to *HSP90AA1* ^-^ *HSP90AB1* ^-^ mast cells (**Figure 6E**). These findings indicated that *HSP90AA1* and *HSP90AB1* might have an unknown mechanism of targeting *SLC25A6* in necroptosis pathway (**Figure 5D**).

### A novel immunological RTM population is specific to paratumor tissue

**Figure 7:**
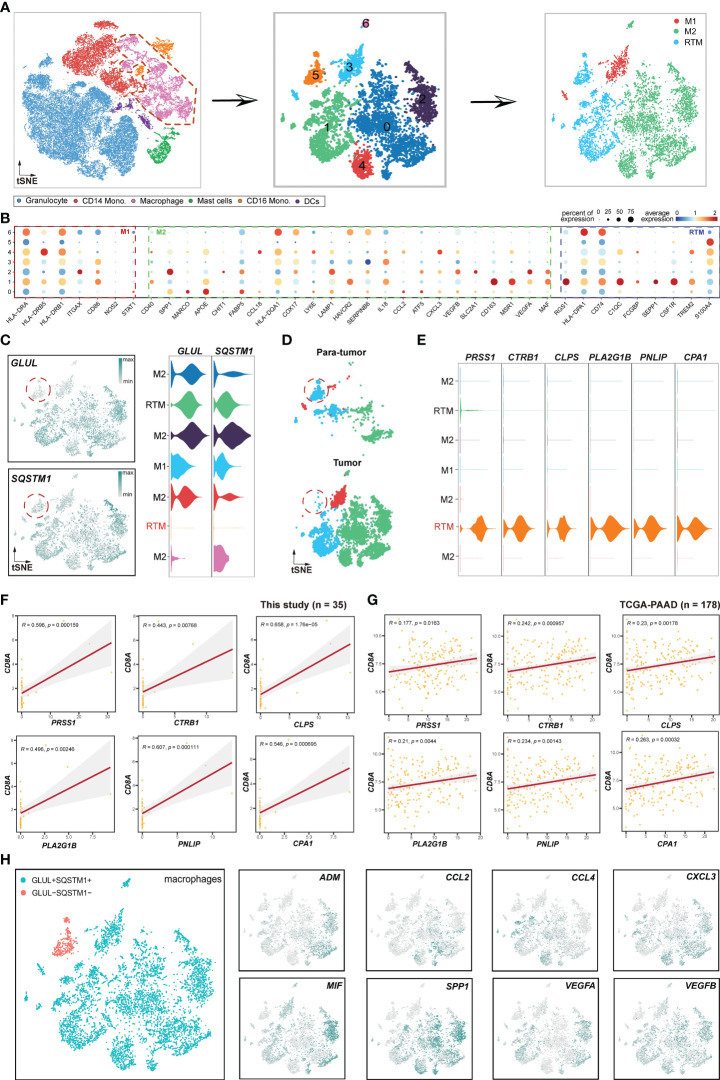
Pipeline to identify novel immunological RTM (resident tissue macrophage) population. **(A)** Schema illustrating the procedures for identifying the subclusters of macrophages. **(B)** Bubble plot displaying selected cell type-specific markers across all clusters. The size of dots represents the fraction of cells expressing a particular marker, and the color intensity indicates the level of average mean expression. **(C)** t-SNE plots showing the expression of *GLUL* and *SQSTM1*. **(D)** t-SNE plots showing the subclusters of macrophages between tumor and paratumor samples. **(E)** Violin plots presenting RTM (*GLUL* ^-^ *SQSTM* ^-^) cluster-specific markers. **(F)** Correlations between the *GLUL* ^-^ *SQSTM1* ^-^ RTM cluster-specific genes and CD8A. **(G)** TCGA validation of the genes illustrated in **(F)**. **(H)** Expression and distribution patterns of cytokines in macrophages. The left figure represents macrophage subsets, while the right figures are expression patterns of some cytokines.

Based on the identified NPA genes mentioned above, we proceeded to investigate the relations within myeloid lineages. Firstly, myeloid cells from tumor and paratumor samples were selected for subsequent analysis (**Figure 7A**, left). Subsequently, an unsupervised clustering analysis was performed on macrophage subsets (**Figure 7A**, middle). Cluster 3, characterized by high expression of *ITGAX*, *CD86*, *HLA-DRA*, and *HLA-DRB1*, was identified as M1 macrophages (**Figure 7B**). Clusters 0, 2, 4, and 6 were classified as M2 macrophages as they highly expressed *SPP1*, *MACRO*, *APOE*, *FABP5*, and *LAMP1*. Clusters 1 and 5, which displayed elevated expression profiles of *S100A4*, *RGS1*, *CD74*, and *CSF1R*,were designated as RTM (resident tissue macrophage) subset.

Focusing on the expression profiles of NPA genes, including *GLUL* and *SQSTM1* in macrophages, strikingly, we found that *GLUL* and *SQSTM1* were elevated in all macrophage subsets except for a minor RTM cell population (**Figure 7C**). Therefore, we named this RMT subset as *GLUL* ^-^ *SQSTM1* ^-^ RTM. Unexpectedly, this subset was deficient in tumor samples compared to paratumor samples (**Figure 7D**), indicating that *GLUL* ^-^ *SQSTM1* ^-^ RTM may act as a positive regulator of immunity. To validate this hypothesis, we performed a correlation analysis of *GLUL* ^-^ *SQSTM1* ^-^ RTM and CD8^+^T cells using *GLUL* ^-^ *SQSTM1* ^-^ RTM markers, including *PRSS1*, *CTRB1*, *CLPS*, *PLA2G1B*, *PNLIP* and *CPA1* (**Figure 7E**). The results showed positive correlations between these markers with CD8+T cells both in this cohort (**Figure 7F**) and TCGA-PAAD cohort (**Figure 7G**), which validated that *GLUL* ^-^ *SQSTM1* ^-^ RTM represents an immunological cell population. Furthermore, to investigate whether this subpopulation directly interacts with epithelial cells, we performed correlation analyses. The results indicated that this subpopulation had no direct correlations with epithelial cells, neither in this cohort (**Figure S7A**) nor in the TCGA-PAAD cohort (**Figure S7B**). This suggests that this subpopulation may not directly exert immunological functions on epithelial cells.

Cytokines, which are small proteins crucial in controlling the growth and activity of the immune system, play a significant role in the immune and inflammatory responses of all cells in the body (47). Alternatively, cancers can respond to host-derived cytokines that promote growth, inhibit apoptosis and facilitate invasion and metastasis (48). In this study, we investigated the expression profiles of several cytokines in macrophages. The results showed that *ADM* (49), *CCL2* (50), *CCL4* (51), *CXCL3* (52), *MIF* (53), *SPP1* (54), *VEGFA* (55) and *VEGFB* (56), which have previously been reported to promote tumor progression and metastasis, were specifically deficient in *GLUL* ^-^ *SQSTM1* ^-^ RTM (**Figure 7H**). This further emphasizes the immunological role of *GLUL* ^-^ *SQSTM1* ^-^ RTM in TME.

### *HSP90AA1* ^+^ *HSP90AB1* ^+^ mast cells are pro-immune

After investigating macrophages, our focus shifted to mast cells (**Figure 6**). We extracted mast cells from the myeloid cell population to perform unsupervised clustering. A total of 6 clusters were identified, and cluster 3 lacked HSP90AA1 and HSP90AB1 expression. Consequently, we termed cluster 3 as *HSP90AA1* ^-^ *HSP90AB1* ^-^ mast cells, while the remaining mast cells were classified as *HSP90AA1* ^+^ *HSP90AB1* ^+^ mast cells (**Figure 6A**). To determine whether *HSP90AA1* ^+^ *HSP90AB1* ^+^ mast cells exhibited pro-immune or anti-immune characteristics, we evaluated the correlations between *HSP90AA1* ^+^ *HSP90AB1* ^+^ mast cell markers (**Figure 6B**) and CD8^+^T cells. The results showed that all of the *HSP90AA1* ^+^ *HSP90AB1* ^+^ mast cell markers, including *HSP90AA1*, *HSP90AB1*, *TPSAB1*, *AREG*, *CPA3*, *JUN*, *LTC4S*, *CLU*, *KIT*, *FAU*, etc., exhibited positive correlations with *CD8A* (**Figures 6C, D**, **S8A**), demonstrating that *HSP90AA1* ^+^ *HSP90AB1* ^+^ mast cell may act as positive regulator of immunity. In contrast, *HSP90AA1* ^-^ *HSP90AB1* ^-^ mast cells were anti-immune, and their markers, including *CLC*, *RUNX1*, *FAM101B*, *SORL1*, *PIM1*, *CSF3R*, *ATP100*, *MAF*, *MYO1F*, etc. (**Figure S8B**), showed negative correlations with CD8^+^ T cells (**Figure S8C**). To further investigate the novel mast clusters, we evaluated the expression patterns of certain cytokines (**Figure 6E**), including *AVP*, *CTSG*, and *NAMPT*. *AVP* (57) and *CTSG* (58), known to play important roles in inflammation and immune responses, were exclusively sufficient in *HSP90AA1* ^+^ *HSP90AB1* ^+^ mast cells, while *NAMPT* (59), previously reported to be associated with maintaining cancer stemness, was highly expressed in *HSP90AA1* ^-^ *HSP90AB1* ^-^ mast cells. Additionally, the expression of *CD8A* was significantly higher in *HSP90AA1* ^+^ *HSP90AB1* ^+^ mast cells (**Figure S8D**), providing further supporting for the notion that *HSP90AA1* ^+^ *HSP90AB1* ^+^ mast cells were immune-promoting.

### *JAK3* ^+^ *TLR4* ^+^ CD16 monocytes are anti-immune

**Figure 8:**
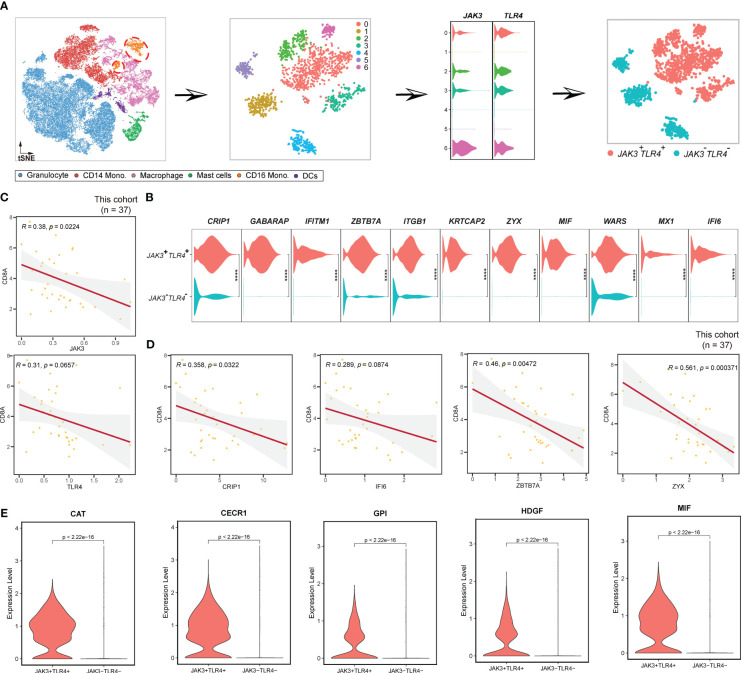
Pipeline to identify *JAK3* ^+^ *TLR4* ^+^ CD16 monocytes. **(A)** Workflow showing the procedures to identify *JAK3* ^+^ *TLR4* ^+^ mast cells. **(B)** Violin plots displaying the expression of *JAK3* ^+^ *TLR4* ^+^ mast cluster-specific genes. **(C)** Scatterplot showing the correlations between *JAK3*, *TLR4*, and *CD8A* in this study. **(D)** Scatterplot illustrating the correlation between *JAK3* ^+^ *TLR4* ^+^ mast cluster-specific genes and CD8A in this study. **(E)** Violin plot showing the expression profiles of some cytokines in CD16 monocytes. **** Represents a statistical significance level of P<0.0001.

Furthermore, we investigated CD16 monocytes (**Figure 8**). Firstly, we extracted CD16 monocytes from myeloid cells and performed unsupervised clustering. As a result, a total of 7 clusters were identified, in which clusters 1, 4, and 5 were all *JAK3* ^-^ *TLR4* ^-^ (**Figure 8A**). Therefore, we classified CD16 monocytes into *JAK3* ^+^ *TLR4* ^+^ CD16 monocytes and *JAK3* ^-^ *TLR4* ^-^ CD16 monocytes (**Figure 8A**). We then proceeded to examine the distinctive features of these two clusters. In contrast to *HSP90AA1* ^+^ *HSP90AB1* ^+^ mast cells, *JAK3* ^+^ *TLR4* ^+^ CD16 monocytes were immunosuppressive, as indicated by the negative correlation between their markers (*JAK3*, *TLR4*, *CRIP1*, *IFI6*, *ZBTB7A*, *ZYX*, etc.) (**Figure 8B**) and CD8^+^T cells at a significant levels (**Figures 8C, D**). In contrast, *JAK3* ^-^ *TLR4* ^-^ CD16 monocytes displayed upregulation of *EEF1D*, *MS4A4A*, *TMEM66*, and TNF (**Figure S9A**), all of which were positively correlated with CD8^+^ T cells at significant levels (**Figure S9B**), suggesting a pro-immune role of *JAK3* ^-^ *TLR4* ^-^ CD16 monocytes. Cytokine analysis further confirmed these results. *CAT* (60), *CECR1* (61), *GPI* (62), *HDGF* (63), and *MIF* (53), previously reported to promote tumor development and progression, were specifically abundant in *JAK3* ^+^ *TLR4* ^+^ CD16 monocytes, rather than *JAK3* ^-^ *TLR4* ^-^ CD16 monocytes (**Figure 8E**).

Furthermore, we explored the NPA genes in other myeloid cells, however, the correlations between these genes and *CD8A* were insignificant. For example, in *BIRC3* ^+^ CD14 monocytes, the marker *BIRC3*, did not show a significantly correlation with CD8^+^ T cells (**Figure S10A**). Moreover, in *BIRC3* ^+^ CD16 monocytes, although *BIRC3* exhibited a significant positive correlation with *CD8A*, other markers such as *APRT*, *C1QB*, *GABARAP*, and *IFITM1* were not correlated with CD8^+^ T cells (**Figure S10B**). Moreover, in *CHMP1B* ^+^ DCs, the marker *CHMP1B* was not correlated with CD8^+^ T cells (**Figure S10C**). These results indicated that these NPA genes may function as immune mediators in an unknown manner.

### Cluster-specific cellular interaction networks that mediate immunity

**Figure 9:**
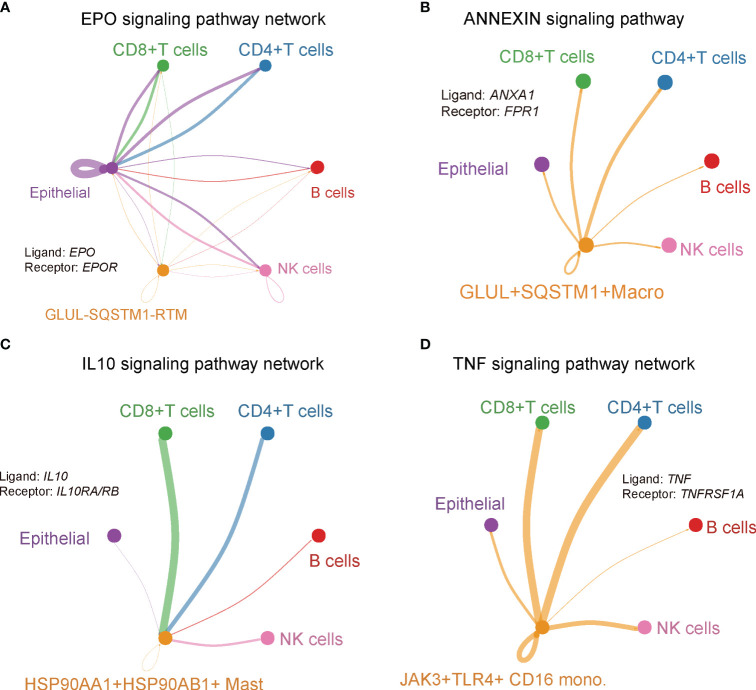
Cluster-specific interaction network. Signaling pathway network of ligand-receptor pairs that were specific in **(A)** *GLUL* ^-^ *SQSTM1* ^-^ RTM, **(B)** *GLUL* ^+^ *SQSTM1* ^+^ macro, **(C)** *HSP90AA1* ^+^ *HSP90AB1* ^+^ mast, **(D)** *JAK3* ^+^ *TLR4* ^+^ CD16 Mono.

**Figure 10:**
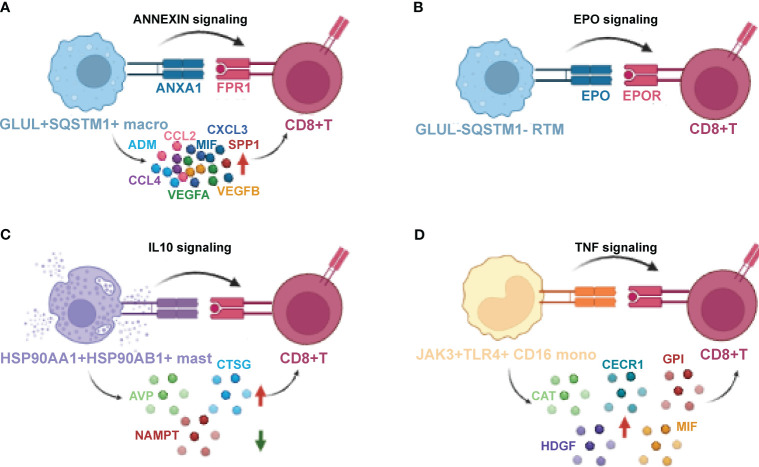
The crosstalk between novel myeloid-derived novel clusters, CD8^+^T cells Macrophage **(A)**, GLUL-SQSTM1-Macrophage **(B)**, HSP90AA1^+^ HSP90AB1^+^ Mast **(C)** and JAK3^+^ TLR4^+^ CD16^+^ Monocyte **(D)**, and CD8^+^T cells that mediate immunity. Schematic for cellular crosstalk and corresponding signaling pathways in PDAC TIMs that contribute to immunity or immune suppression. The novel clusters secrete various cytokines and ligands that signal to their respective receptors, thus activating the corresponding signaling, respectively. Red arrows indicate up-regulated cytokines while green arrow indicates down-regulated cytokines.

To gain a comprehensive understanding of the interactions among the novel clusters and the T/B/NK/epithelial cell populations, as well as their collective contribution to the PDAC tumor microenvironment, we inferred a putative cellular interaction network based on the receptor-ligand database (31). Our findings unveiled specific interactions within various signaling pathways. Specifically, we observed that *EPO/EPOR* interaction of EPO signaling pathway was unique to the *GLUL* ^-^ *SQSTM1* ^-^ RTM cluster. In this cluster, the ligand EPO was predominantly expressed in *GLUL* ^-^ *SQSTM1* ^-^ RTM, while *EPOR* receptor was present in CD4^+^ T cells, CD8^+^ T cells, B cells, NK cells and epithelial cells (**Figure 9A**). The *EPO/EPOR* interaction has been reported to initiate a signaling cascade that activated and recruited a variety of Src homology-2 (SH2) domain-containing proteins, subsequently triggering downstream signaling pathways such as ERK-1/2 and JAK-2 (64). Interestingly, a recent study has demonstrated that *EPO/EPOR* could reduce the variability of myeloma cell lines and malignant primary plasma cells (65). And *ANXA1/FPR1* interaction within the ANNEXIN signaling pathway was specific to *GLUL* ^+^ *SQSTM1* ^+^ macrophage cells, and ligand *ANXA1* was specific to *GLUL* ^+^ *SQSTM1* ^+^ macrophage, while receptor *FPR1* was in CD4^+^ T, CD8^+^ T, B, NK and epithelial cells (**Figure 9B**). *FPR1*, previously reported to promote chemotherapy-induced antitumor immune response (66), was demonstrated to act as a receptor for *ANXA1*, promoting cell death through the necroptosis pathway (67). Furthermore, we identified the *IL10/IL10RA* interaction within the IL10 signaling pathway, which was specific to *HSP90AA1* ^+^ *HSP90AB1* ^+^ mast cell, in which ligand *IL10* was expressed in CD4^+^ T, CD8^+^ T, B, NK, and epithelial cells, while the receptor *IL10RA* was specific to *HSP90AA1* ^+^ *HSP90AB1* ^+^ mast cell (**Figure 9C**). IL10- and IL10R-dependent signaling have been reported to play critical roles in controlling immune responses in both innate and adaptive immune systems (68). Finally, TNF-α (TNF)/TNFR1 (*TNFRSF1A*) interaction was found to be specific to *JAK3* ^+^ *TLR4* ^+^ CD16 monocytes. In this interaction, the ligand TNF was specific to *JAK3* ^+^ *TLR4* ^+^ CD16 monocytes, while receptor *TNFR1* was expressed in CD4^+^ T, CD8^+^ T, B, NK and epithelial cells (**Figure 9D**). TNF-α signaling meditated by *TNFR1* in the TME has been reported to promote gastric tumor development and maintain tumor cells in an undifferentiated state (69). Taken together, the intercellular interactions revealed a close relationship between immune cell and cancer cell dynamics, as well as the molecular features of novel clusters (**Figure 10**). These interactions may play a crucial role in determining the prognostic and therapeutic response in PDAC.

### Validation of the existence of the novel clusters

**Figure 11:**
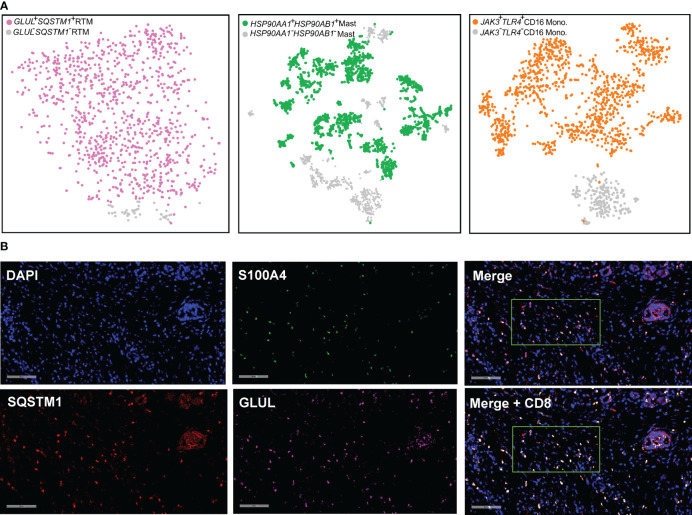
Detection and validation of the cellular clusters. **(A)** t-SNE plot combing *GLUL* ^+^ *SQSTM1* ^+^ RTM (n = 998, in purple), *HSP90AA1* ^+^ *HSP90AB1* ^+^ mast cells (n = 2,151, in blue), and *JAK3* ^+^ *TLR4* ^+^ CD16 monocyte (n = 1,234, in orange) from PDAC (Peng et al., 2019). **(B)** RNA FISH staining in the PDAC tissues. One representative image for each gene is shown. Spectrum orange dots indicate GLUL, spectrum red dots indicate SQSTM1, spectrum green dots indicate S100A4, spectrum gold indicate CD8A. Scale bars, 90 μm.

We proceeded to validate the presence of *GLUL* ^-^ *SQSTM1* ^-^ RTM, *HSP90AA1* ^+^ *HSP90AB1* ^+^ mast cell, and *JAK3* ^+^ *TLR4* ^+^ CD16 monocyte cellular clusters in other PDAC cohorts. To accomplish this, we analyzed publicly available scRNA-seq data from the CRA001160 dataset (26). In this dataset, 1,047, 3,098, and 1,464 cells were annotated as RTM, mast cells, and CD16 monocytes, respectively (**Figure S1**). To focus specifically on RTM, mast cells, and CD16 monocytes, we distinguished these cell types based on the expression of *GLUL*/*SQSTM1* (RTM), *HSP90AA1*/*HSP90AB1* (mast cell), and *JAK3*/*TLR4* (CD16 monocytes), respectively (**Figure 11A**). Consequently, we obtained 49 *GLUL* ^-^ *SQSMT1* ^-^ RTM, 2,151 *HSP90AA1* ^+^ *HSP90AB1* ^+^ mast cells, and 1,234 *JAK3* ^+^ *TLR4* ^+^ CD16 monocytes (**Figure 11A**).

Although GLUL^-^SQSMT1^-^ RTM was specifically present in PDAC tumor tissues, it was noteworthy that the majority of RTM in the TME are characterized by the expression of GLUL and SQSTM1. Given the prevalence of GLUL^+^SQSTM1^+^RTM cells in PDAC tumor tissues, and their interaction with CD8^+^T cells revealed by the cell chat analysis, it becomes apparent that a deeper exploration is warranted. To validate the expression of these genes at single-cell level, visualize their spatial distribution within complex tissue structures and validate co-expression patterns, functional gene interactions, we performed RNA fluorescence *in situ* hybridization (FISH) on paraffin sections of PDAC tumor tissue (**Figure 11B**).Our analysis revealed frequent overlap of the *GLUL*, *SQSTM1* and *S100A4*, providing evidence for the existence of the GLUL^+^SQSTM1^+^ RTM. Additionally, we conducted further investigations and confirmed the adjacent spatial relationship between the GLUL^+^SQSTM1^+^ RTM and CD8^+^ T cells in PDAC solid tumors. This observation suggests potential functional interactions between these cell populations within the tumor microenvironment.

## Discussion

It is well-established that necroptosis, a programmed form of necrosis or inflammatory cell death (70), has gained significant attention in cancer research due to its implications in pathogenesis and therapy (40, 71, 72). The involvement of necroptosis in recruiting immune cells, regulating pro- or anti-tumor components in TME, and modulating immune responses through the release of DAMPs, chemokines and other cytokines. However, the specific roles of necroptosis in PDAC have not been systematically described and remain to be deciphered. In this study, public scRNA-seq data from Gene Expression Omnibus (GEO) database was downloaded, which covered more than 124,000 cells of 16 PDAC patients across multiple immune-relevant tissue sites (24). Our analysis focused on charactering major myeloid cell lineages, and identifying three necroptosis-associated subsets of myeloid-derived cells. These findings provide a valuable resource for comprehensively understanding multi-dimensional characterization of the tumor microenvironment in PDAC.

In this study, we identified *HSP90AA1* ^+^ *HSP90AB1* ^+^ mast cells that exerting anti-tumorigenic effects in PDAC, contributing to better clinical outcomes. the Heat shock protein 90 (HSP90) protein family, including *HSP90AA1* and *HSP90AB1*, plays prominent roles in various biological processes such as protein folding (73), apoptosis (74), cell-cycle regulation (75), as well as signal transduction (76). Of note, previous studies have suggested that HSP90 can influence the activation and stability of crucial regulators involved in the necroptosis process, such asRIPK1, RIPK3 and MLKL, thereby contributing to immune cell recruitment and immunogenic cell death of tumor cells (77–79). In addition, necroptosis is deemed to trigger an adaptive immune response by releasing cytokines (80). The novel mast cells were predicted to interact with CD8^+^ T cells via IL10 signaling pathway, and highly expressed *AVP* and *CTSG*. Consistently, *AVP* (57) and *CTSG* (58) have been reported to play important roles in inflammation and immune response, and IL10 has been shown to potentiate IFN-γ and induct the cytotoxicity of CD8^+^ T cells (68, 81–83), thereby triggering anti-tumor immune responses. These findings collectively suggested that *HSP90AA1* ^+^ *HSP90AB1* ^+^ mast cells are functionally important in necroptosis process and are involved in immune cell recruitment through the IL10 signaling pathway. Future research should focus on designing effective drugs that modulate HSP90 activity and developing diagnostic tools for accurate patient stratification for therapy with HSP90 agonists or HSP90 antagonist.

Another cluster of special interest is the *GLUL* ^-^ *SQSTM1* ^-^ RTM subpopulation, which is enriched in PDAC tumor tissues and acts as a positive regulator of immunity. Glutamine synthetase (GLUL) has been associated with RIP3-mediated necroptosis (41), and the p62/SQSTM1 complex binding to necroptosis-related proteins RIP1 and RIP3 facilitates the transition from autophagy to necroptosis (84). Moreover, it has been reported that both GLUL and p62/SQSTM1 may influence the recruitment, activation and polarization of macrophage. *GLUL* is known to be associated with the differentiation and function of macrophage, more specifically, enhancing M2- polarization (85, 86). Exogenous p62/SQSTM1 has been shown to induce M1 polarization of macrophage through activation of the NF-κB pathway (87). Given the potential roles of GLUL and p62/SQSTM1 in macrophage polarization, we evaluating the expression levels of molecular markers in macrophages. For example, CCL2/CCR2 axis is a major player in macrophage polarizing towards to M2 phenotype (88, 89). Stimulation of CXCL13 may activate Akt pathway, suggesting an increase in M2 macrophage in renal cell carcinoma (90). The vascular endothelial growth factor (VEGF) family, including *VEGFA* and *VEGFB*, can contribute to M2 polarization in the decidua (91). *MIF* (92), *SPP1* (93) and *ADM* (94) have all been previously reported to be associated with a dominant M2 polarization and a loss of M1 function. In our study, the deficiency of these chemokines in *GLUL* ^-^ *SQSMT1* ^-^ RTM cells may suggest the pro-inflammatory M1-polarized phenotype, thereby modulating the antitumor response. These results indicated that the GLUL^-^SQSMT1^-^ RTM cells with a pro-immune profile may evade programmed necroptosis and abundantly infiltrate in the PDAC TME of patients with superior efficacy. The combination of *GLUL* and *SQSTM1* inhibitors in precisely characterized patients may have superior effects against cancer compared to immunotherapy alone.

The novel *JAK3* ^+^ *TLR4* ^+^ CD16 monocyte subset exhibits anti-immune properties and is associated with unfavorable clinical outcomes. Janus Kinase 3 (*JAK3*) is a tyrosine kinase that belongs to the Janus family of kinases. Hyper-activation of the JAK3-STAT signaling pathway has been linked to tumor development and progression by inducing factors associated with suppressive immune cell recruitment, angiogenesis and neo-vascularization (95–99). TLR4, a member of the toll-like receptor (TLR) family, can lead to the activation of NF-κB pathway, which is essential for necroptosis signaling, as well as the production of pro-inflammatory cytokines and angiogenetic factors (100, 101). In addition, we observed that this novel monocyte subset was predicted to interact with CD8^+^ T cells via TNF-TNFR1 signaling pathway and expressed high levels of pro-tumor cytokines CAT, HDGF, CECER1, GPI and MIF. NFR1-dependent TNF signaling has been reported to impair the accumulation of tumor-infiltrating lymphocyte (TILs) and induce significant death of activated CD8^+^ T cells (102, 103). HDGF is considered as an angiogenic and anti-apoptotic factor, contributing to tumorigenesis in several malignant diseases (104–106). MIF plays an essential role in inhibiting cytotoxic T lymphocytes (CTLs) and regulating lymphocyte transmigration (107, 108). CAT, CECR1 and GPI have all been previously associated with promoting tumor progression (60–62). Altogether, these results align with our observations. We hypothesized that this monocyte subset (*JAK3* ^+^ *TLR4* ^+^ CD16 monocyte) contributes to shaping pro-tumor immunity in TME, ultimately accelerating malignant transformation and tumor progression. However, further investigation is needed to elucidate the underlying mechanisms. Compared to other JAKs, *JAK3* has a more restricted expression profile, primarily confined to immune system. Therefore, selective targeting of *JAK3* represents a potent immunosuppressant strategy that could minimize potential adverse effects. Inhibition of TLR4-related pathways has shown promising results in clinical trials for disease treatment with excessive immune response (109–111). Current study supports the notion that the discovery of *JAK3* and *TLR4* antagonists could be an ideal strategy for cancer treatment.

A major limitation of the current finding is the lack of sufficient experimental validation. For instance, the proposed NPA gene network was supposed only based on the expression profiles, and the underlying regulatory mechanism of necroptosis pathway in tumor progression remains obscure in real world. In addition, our study illustrated the indispensable roles of three novel myeloid subpopulations in tumor microenvironment and their associations with necroptosis, however, the underlying mechanisms need further investigation. And the expression profiles of GLUL and SQSTM1 of macrophages in normal samples were not explored. The implementation of advanced biological techniques and bioinformatics analysis in mammalian models of human pathological samples will be critical for gaining a better understanding of these subpopulations in the context of molecular mechanism and drug targeting.

Given the robust immunosuppressive and desmoplastic TME in PDAC, which contributes to adaptive or acquired resistance to therapy, investigating the relationship between necroptosis and tumor immunology holds promise for future treatment solutions. The identification of necroptosis-associated myeloid lineages can potentially serve as targets for therapeutic intervention, allowing for dynamically monitoring of the anti-tumor immune response and improvement of patient outcomes. Our findings provide a valuable resource for further investigation to gain deeper biological insights into the role of necroptosis in cancer. Considering the exceedingly complex and individually unique immune microenvironment of tumors, necroptosis signaling may generate a diverse array of inflammatory responses, ranging from facilitation of anti-tumor to pro-tumor signaling. The three novel necroptosis-associated myeloid subpopulations uncovered in our research may communicate with other cells to mediate ECM degradation and remodeling, signaling pathway regulation and immune cell polarization. These cells and their respective products hold potential as therapeutic targets in PDAC and other types of cancers, enabling the establishment of effective necroptosis-based cancer therapy regimens.

## Data availability statement

The datasets presented in this study can be found in online repositories. The names of the repository/repositories and accession number(s) can be found in the article/**Supplementary Material**.

## Ethics statement

The studies involving humans were approved by The Forth Medical Center of PLA General Hospital. The studies were conducted in accordance with the local legislation and institutional requirements. Written informed consent for participation in this study was provided by the participants’ legal guardians/next of kin. Written informed consent was obtained from the individual(s) for the publication of any potentially identifiable images or data included in this article.

## Author contributions

HXZ: Conceptualization, Methodology, Writing – original draft. WD: Conceptualization, Methodology, Writing – original draft. SX: Writing – original draft, Data curation, Software, Visualization. LZ: Data curation, Software, Visualization, Writing – original draft. TF: Data curation, Software, Visualization, Writing – original draft. LW: Writing – original draft, Investigation. HZ: Writing – review & editing. YH: Writing – review & editing. JY: Writing – review & editing. TW: Writing – review & editing, Conceptualization, Methodology, Resources, Supervision. WX: Conceptualization, Methodology, Resources, Supervision, Writing – review & editing.

## Supplementary material

Supplementary Figure 1 Heterogeneity of TME in PDAC. **(A)** t-SNE plots of cells from 39 samples profiled in this study. Colored by cell types. **(B)** The proportion of 11 cell types among primary tumors, metastatic tumors and paratumor samples, respectively. **(C)** The histogram of 11 cell types among clinical-stage I-IV. **(D)** The percentage of cell types between PBMCs from PDAC Patients and PBMCs from healthy controls. **(E)** The proportion of 11 cell types compared between primary tissues and metastatic tumor tissues of PDAC patients with PBMC samples from PDAC patients.

Supplementary Figure 2 Functional analysis of B cells in PDAC. **(A)** Top 100 genes were used to calculate the cox coefficient for each cluster using TCGA (PAAD, n = 183) data. **(B)** t-SNE plots showing the expression of *BCL11A* and *DNASE1L3*. **(C)** Scatterplots showing the correlation between *BCL11A*, *DNASE1L3*, and *CD8A* using TCGA (PAAD, n = 183) data. **(D)** The body maps showing the expression of *BCL11A* and *DNASE1L3* between tumor and normal samples via GEPIA 2 (http://gepia2.cancer-pku.cn/#index). **(E)** Kaplan-Meier overall survival analysis of the high and low groups of *BCL11A* (top) and *DNASE1L3* (bottom). **(F)** DFS (Disease-Free Survival) analysis of the high and low groups of *BCL11A* (top) and *DNASE1L3* (bottom). The hazard ratio was calculated based on Cox PH Model, and 95% CI (Confidence Interval) was applied.

Supplementary Figure 3 Differential gene and pathway analysis for peripheral blood. **(A)** t-SNE plots of myeloid lineages of PBMC from healthy individuals and PDAC patients. **(B)** Proportion of each myeloid cell lineage in PBMC samples from PDAC patients and healthy controls. **(C)** Tissue prevalence estimated by Ro/e score of primary tumor/metastatic tumor/paratumor tissues from PDAC patients (left), tissue/PBMC (middle) from PDAC patient, and PBMCs from PDAC patient/healthy controls (right). **(D)** Top 10 differential up- or down-regulated genes across myeloid lineages of PBMCs from PDAC patient and normal samples. Red dots indicate statistically significant genes (adjusted p-value < 0.01). **(E–I)** Pathways enriched by DEGs of each cell type between PBMCs from PDAC patients and PBMCs from healthy controls.

Supplementary Figure 4 Differential genes and pathways between tumor and paratumor tissue of PDAC. **(A)** Differential gene expression analysis shows up- and down-regulated genes across all cell types between tumor and paratumor tissue from PDAC patients. The top 10 DE genes were shown, and the points dotted in red indicate significant genes. An adjusted p-value < 0.01 is indicated in red, while an adjusted p-value ≥ 0.01 is indicated in black. **(B–F)** Differential pathway enriched in tumor and paratumor from PDAC tissue for each cell type.

Supplementary Figure 5 Differential genes and pathways between PDAC tissue and peripheral blood. **(A)** Differential gene expression analysis showing up- and down-regulated genes across all cell types between tumor tissue samples and PBMC from PDAC patients. The top 10 DE genes were shown, and the points dotted in red indicate significant genes. An adjusted p-value < 0.01 is indicated in red, while an adjusted p-value ≥ 0.01 is indicated in black. **(B–E)** Differential pathway enriched in tissue and PBMC from PDAC patients for each cell type.

Supplementary Figure 6 Visualization of overlapped necroptosis-associated DE genes. Violin plots showing overlapped up-regulated **(A)** and down-regulated **(B)** necroptosis-associated genes (denoted in ) in tumor. *P < 0.05, **P < 0.01, ***P < 0.001.

Supplementary Figure 7 The novel immunological RTM has no direct correlation with epithelial cells. **(A)** Scatterplots showing the correlation between the expression of GLUL^-^SQSTM1^-^RTM cluster-specific genes (denoted in ) and EPCAM. **(B)** TCGA validation of the correlations. Data are from TCGA-PAAD (n = 178).

Supplementary Figure 8
*HSP90AA1* ^-^ *HSP90AB1* ^-^ mast cells are anti-immune. **(A)** Scatterplot showing the correlations between the rest of *HSP90AA1* ^+^ *HSP90AB1* ^+^ mast cluster-specific genes (shown in ) and CD8A in this study. **(B)** Violin plots showing the expression of *HSP90AA1* ^-^ *HSP90AB* ^-^ mast cluster-specific genes. **(C)** Scatterplots showing the correlations between *HSP90AA1* ^-^ *HSP90AB1* ^-^ mast cluster-specific genes and *CD8A* in this study. **(D)** Expression profiles of CD8A in *HSP90AA1* ^+^ *HSP90AB1* ^+^ mast cells vs. *HSP90AA1* ^-^ *HSP90AB1* ^-^ mast cells.

Supplementary Figure 9 JAK3^-^TLR4^-^ CD16 monocytes are pro-immune. **(A)** Violin plots showing the expression of *JAK3* ^-^ *TLR4* ^-^ mast cluster-specific high expression genes. **(B)** Scatterplots showing the correlations between the expression of *JAK3*, *TLR4*, and *CD8A* in this study.

Supplementary Figure 10 Several myeloid subsets may act as pro/anti-immune regulators in a non-necroptosis way. Workflow showing the procedures to distinguish CD14 monocyte **(A)**, CD16 monocyte **(B)**, and DCs **(C)** by *BIRC3*, *PPIA*, and *CHMP1B*, respectively.
