# Supplementary figures and images for: Single-cell RNA-seq analyses inform necroptosis-associated myeloid lineages influence the immune landscape of pancreas cancer
Source: Front Immunol. 2023 Dec 12;14:1263633. doi: 10.3389/fimmu.2023.1263633 (PMC10749962; doi:10.3389/fimmu.2023.1263633)

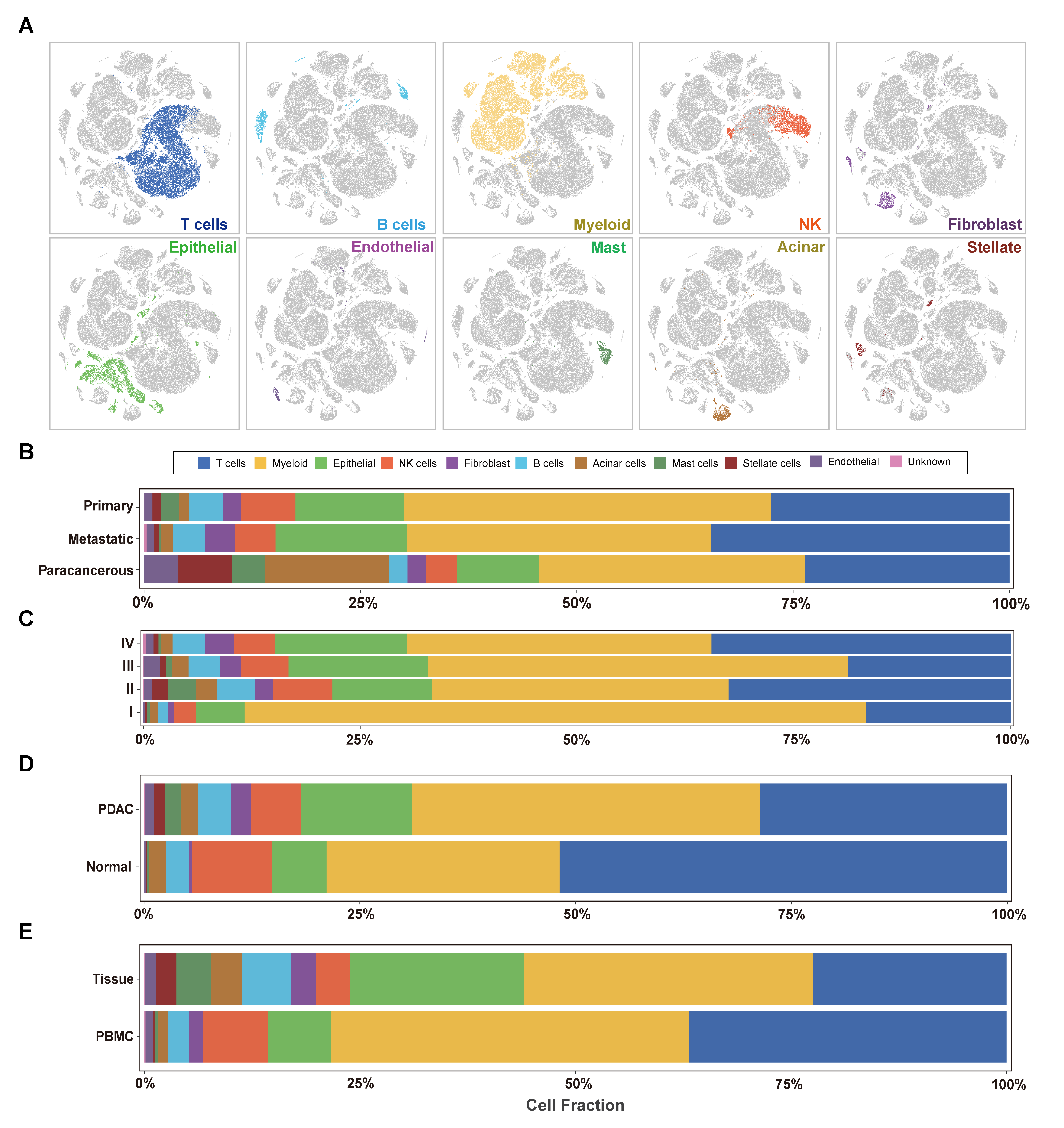

Supplement: Supplementary Figure 1 — Heterogeneity of TME in PDAC. (A) t-SNE plots of cells from 39 samples profiled in this study. Colored by cell types. (B) The proportion of 11 cell types among primary tumors, metastatic tumors and paratumor samples, respectively. (C) The histogram of 11 cell types among clinical-stage I-IV. (D) The percentage of cell types between PBMCs from PDAC Patients and PBMCs from healthy controls. (E) The proportion of 11 cell types compared between primary tissues and metastatic tumor tissues of PDAC patients with PBMC samples from PDAC patients. [file Image_1.tif]

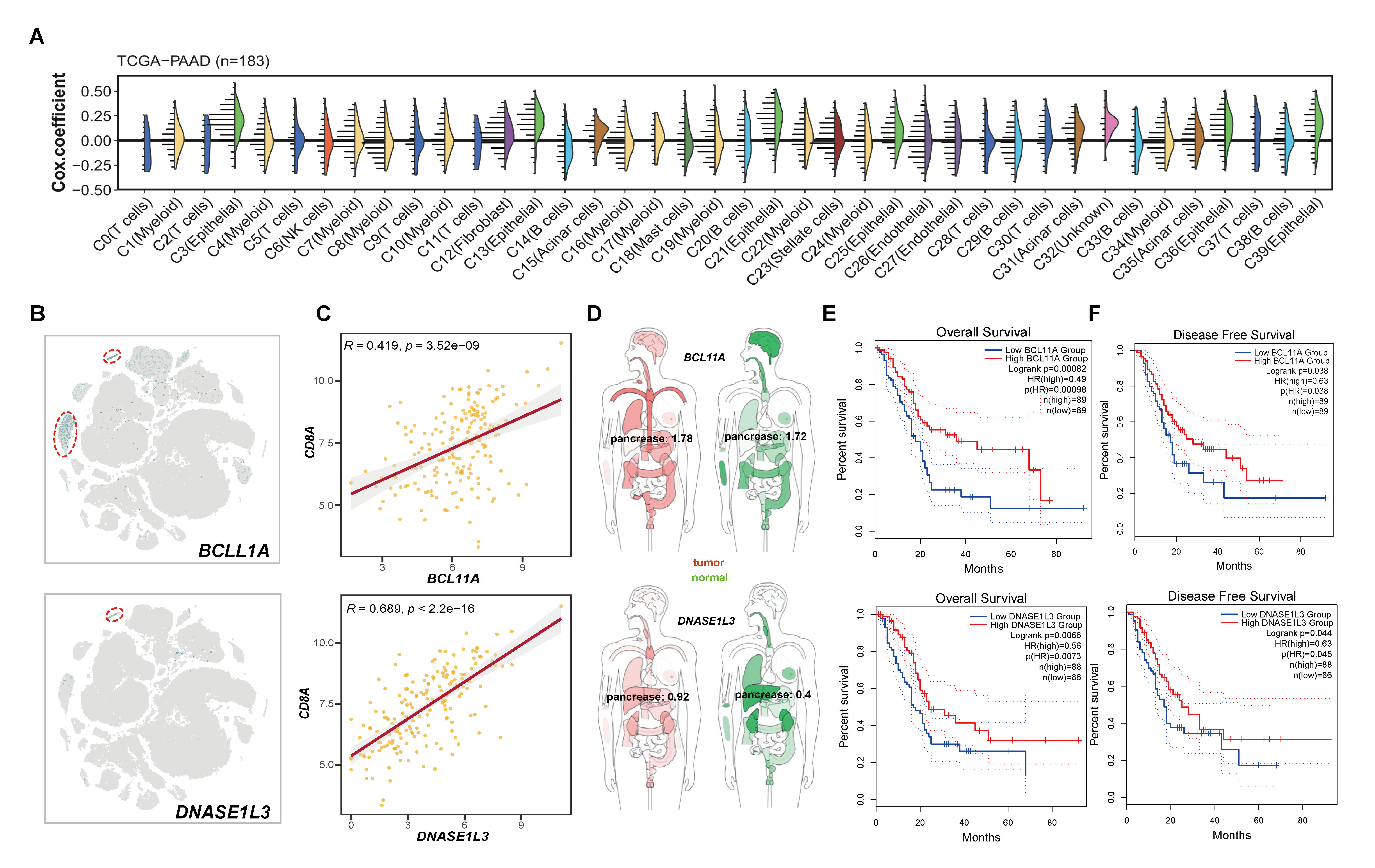

Supplement: Supplementary Figure 2 — Functional analysis of B cells in PDAC. (A) Top 100 genes were used to calculate the cox coefficient for each cluster using TCGA (PAAD, n = 183) data. (B) t-SNE plots showing the expression of BCL11A and DNASE1L3. (C) Scatterplots showing the correlation between BCL11A, DNASE1L3, and CD8A using TCGA (PAAD, n = 183) data. (D) The body maps showing the expression of BCL11A and DNASE1L3 between tumor and normal samples via GEPIA 2 (http://gepia2.cancer-pku.cn/#index). (E) Kaplan-Meier overall survival analysis of the high and low groups of BCL11A (top) and DNASE1L3 (bottom). (F) DFS (Disease-Free Survival) analysis of the high and low groups of BCL11A (top) and DNASE1L3 (bottom). The hazard ratio was calculated based on Cox PH Model, and 95% CI (Confidence Interval) was applied. [file Image_2.tif]

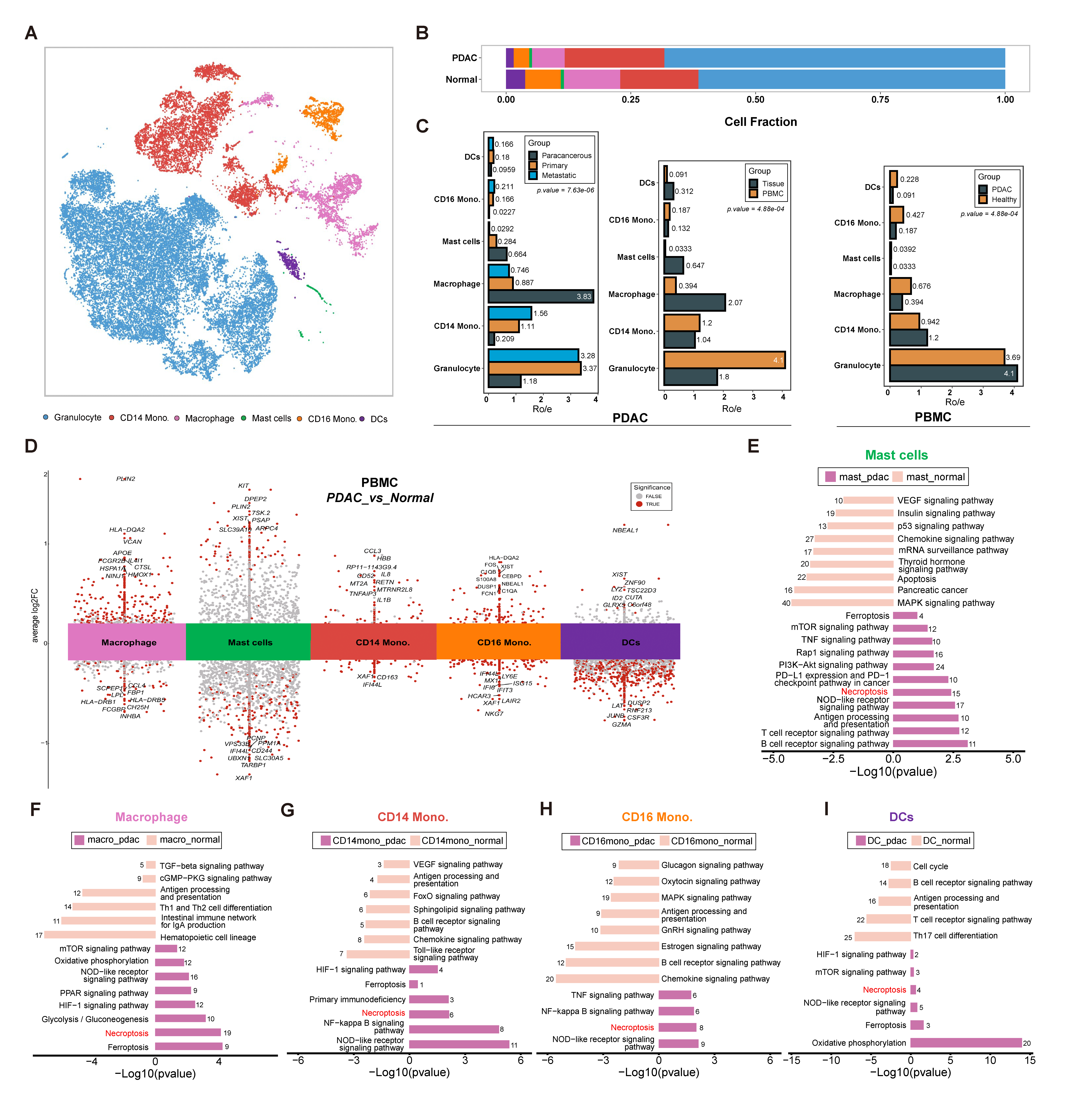

Supplement: Supplementary Figure 3 — Differential gene and pathway analysis for peripheral blood. (A) t-SNE plots of myeloid lineages of PBMC from healthy individuals and PDAC patients. (B) Proportion of each myeloid cell lineage in PBMC samples from PDAC patients and healthy controls. (C) Tissue prevalence estimated by Ro/e score of primary tumor/metastatic tumor/paratumor tissues from PDAC patients (left), tissue/PBMC (middle) from PDAC patient, and PBMCs from PDAC patient/healthy controls (right). (D) Top 10 differential up- or down-regulated genes across myeloid lineages of PBMCs from PDAC patient and normal samples. Red dots indicate statistically significant genes (adjusted p-value < 0.01). (E–I) Pathways enriched by DEGs of each cell type between PBMCs from PDAC patients and PBMCs from healthy controls. [file Image_3.tif]

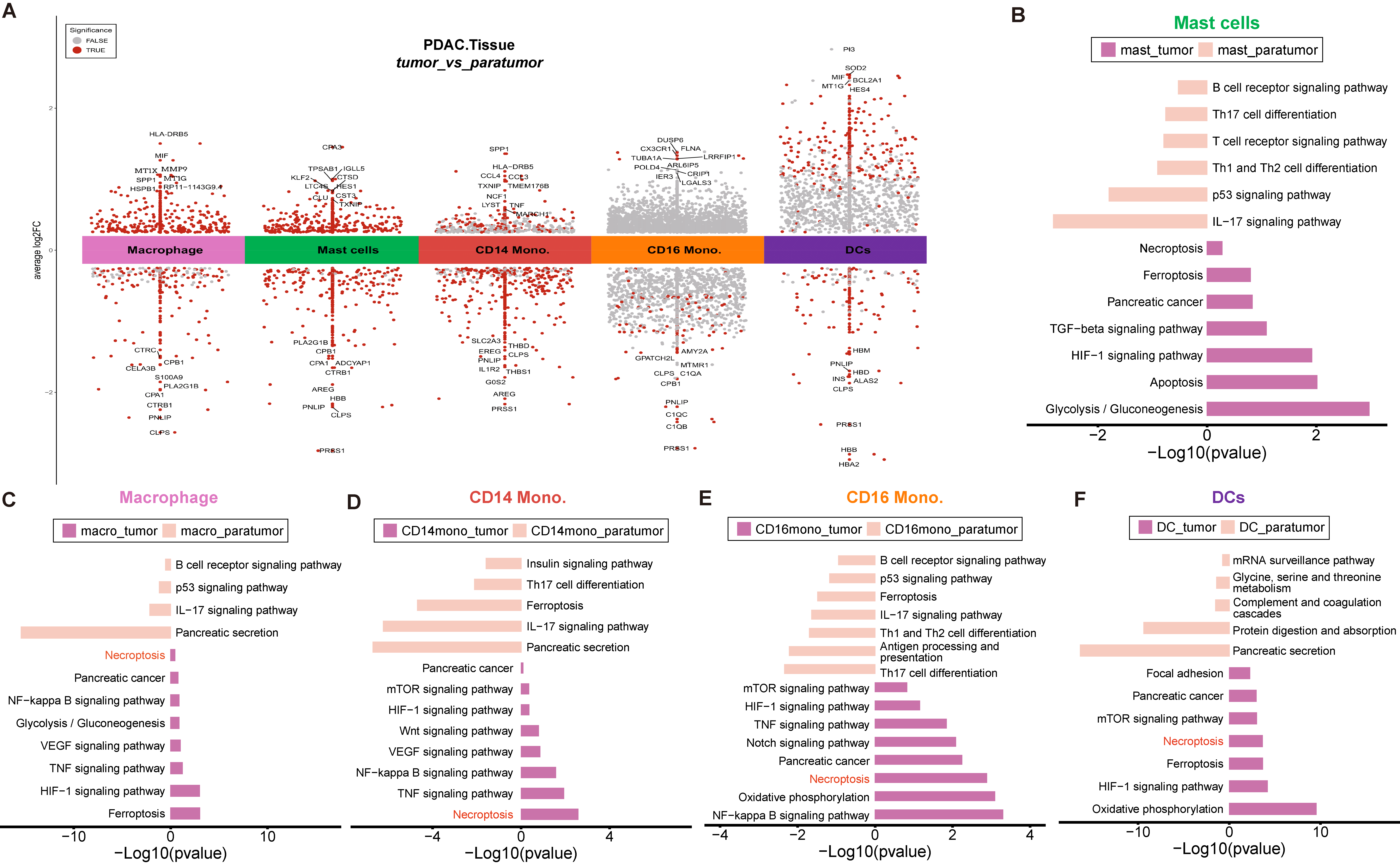

Supplement: Supplementary Figure 4 — Differential genes and pathways between tumor and paratumor tissue of PDAC. (A) Differential gene expression analysis shows up- and down-regulated genes across all cell types between tumor and paratumor tissue from PDAC patients. The top 10 DE genes were shown, and the points dotted in red indicate significant genes. An adjusted p-value < 0.01 is indicated in red, while an adjusted p-value ≥ 0.01 is indicated in black. (B–F) Differential pathway enriched in tumor and paratumor from PDAC tissue for each cell type. [file Image_4.tif]

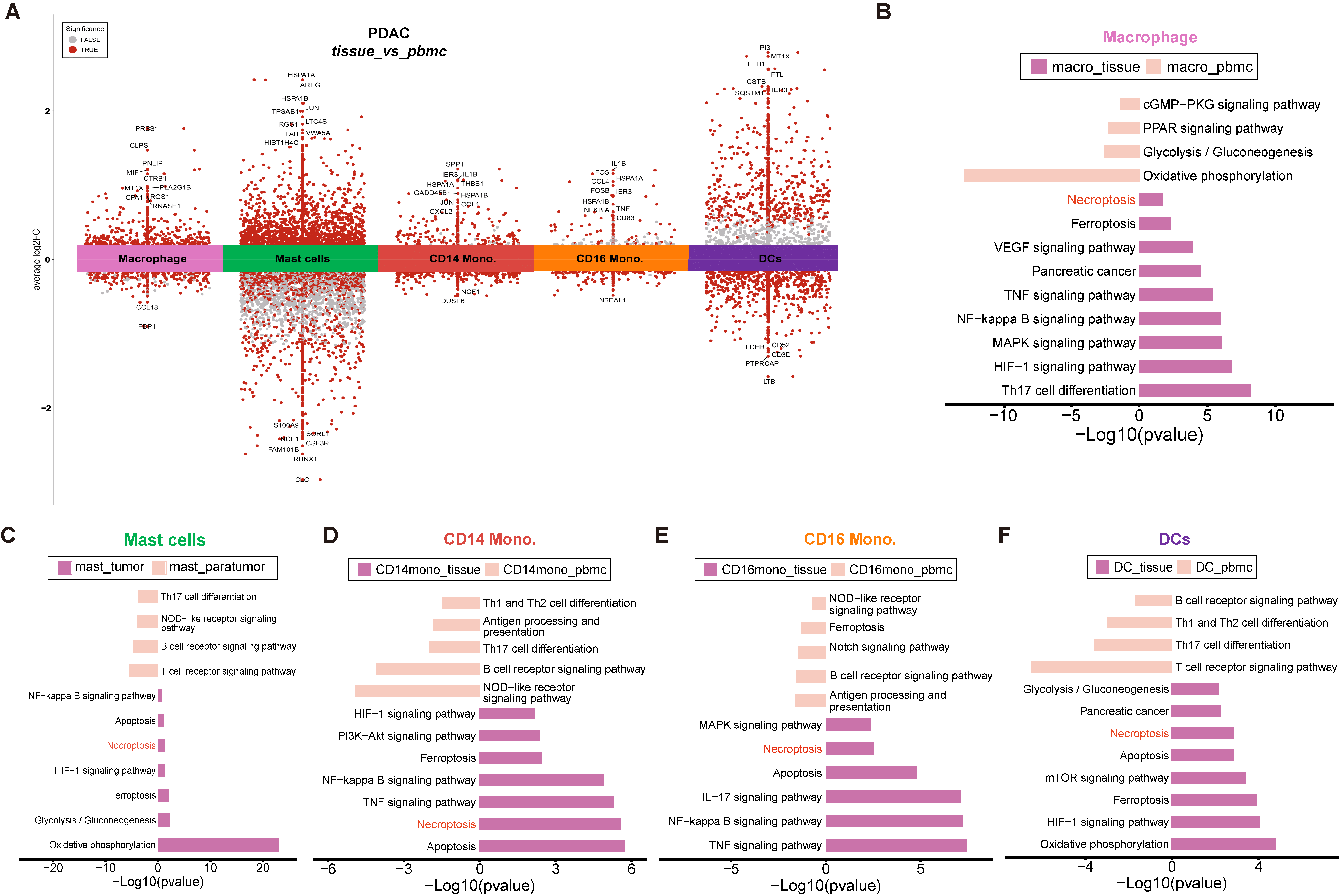

Supplement: Supplementary Figure 5 — Differential genes and pathways between PDAC tissue and peripheral blood. (A) Differential gene expression analysis showing up- and down-regulated genes across all cell types between tumor tissue samples and PBMC from PDAC patients. The top 10 DE genes were shown, and the points dotted in red indicate significant genes. An adjusted p-value < 0.01 is indicated in red, while an adjusted p-value ≥ 0.01 is indicated in black. (B–E) Differential pathway enriched in tissue and PBMC from PDAC patients for each cell type. [file Image_5.tif]

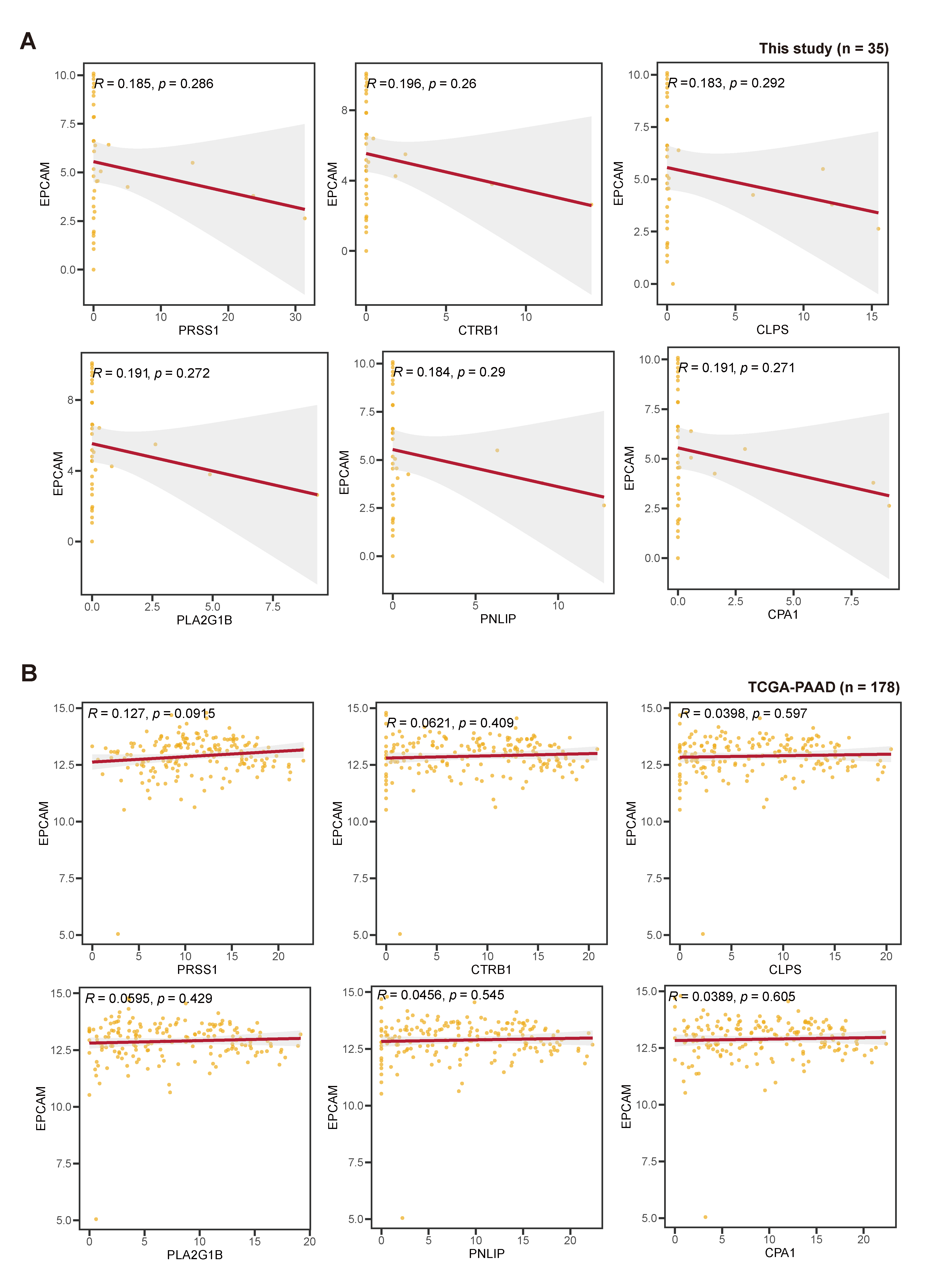

Supplement: Supplementary Figure 7 — The novel immunological RTM has no direct correlation with epithelial cells. (A) Scatterplots showing the correlation between the expression of GLUL-SQSTM1-RTM cluster-specific genes (denoted in ) and EPCAM. (B) TCGA validation of the correlations. Data are from TCGA-PAAD (n = 178). [file Image_7.tif]

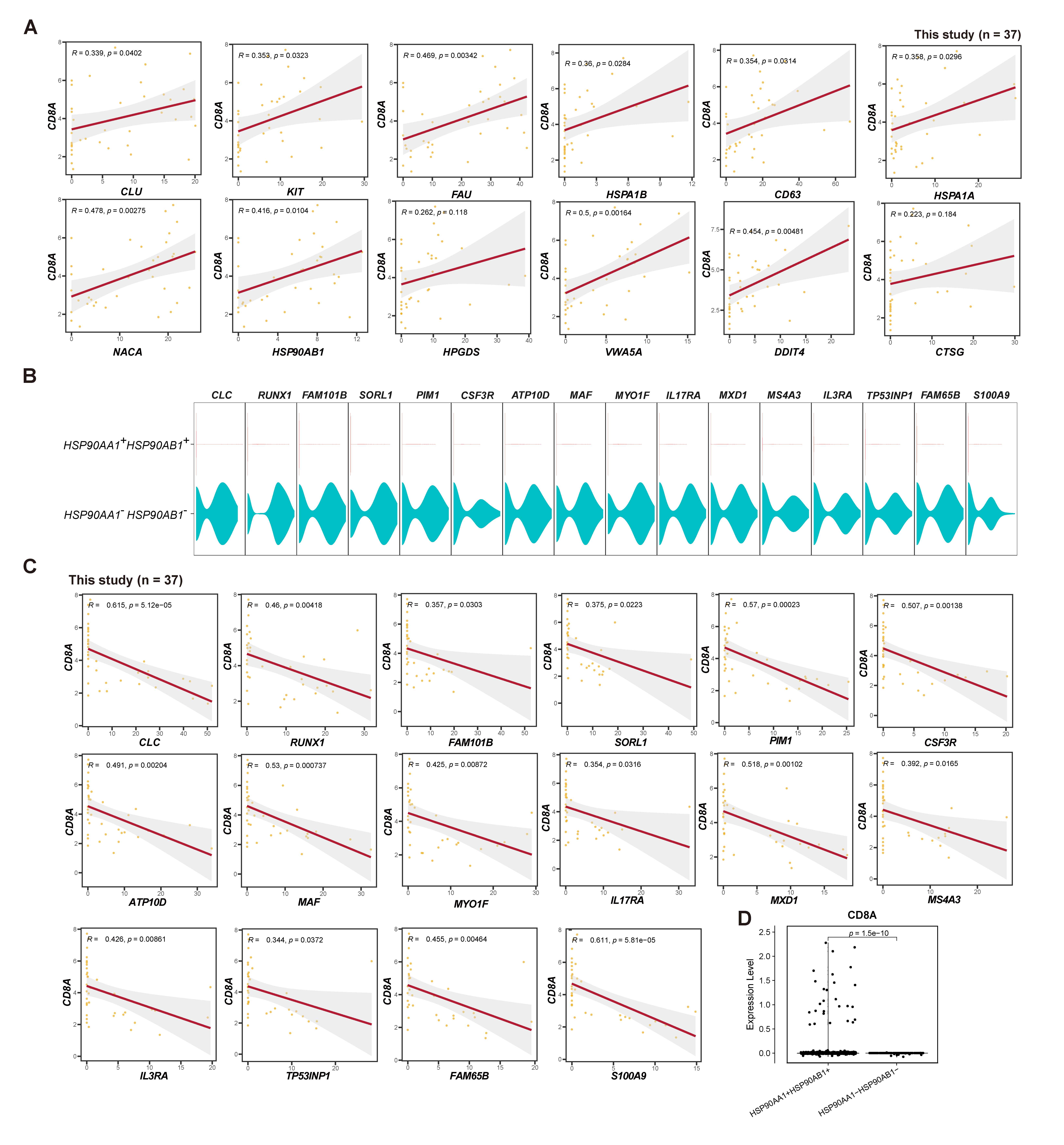

Supplement: Supplementary Figure 8 — HSP90AA1 - HSP90AB1 - mast cells are anti-immune. (A) Scatterplot showing the correlations between the rest of HSP90AA1 + HSP90AB1 + mast cluster-specific genes (shown in ) and CD8A in this study. (B) Violin plots showing the expression of HSP90AA1 - HSP90AB - mast cluster-specific genes. (C) Scatterplots showing the correlations between HSP90AA1 - HSP90AB1 - mast cluster-specific genes and CD8A in this study. (D) Expression profiles of CD8A in HSP90AA1 + HSP90AB1 + mast cells vs. HSP90AA1 - HSP90AB1 - mast cells. [file Image_8.tif]

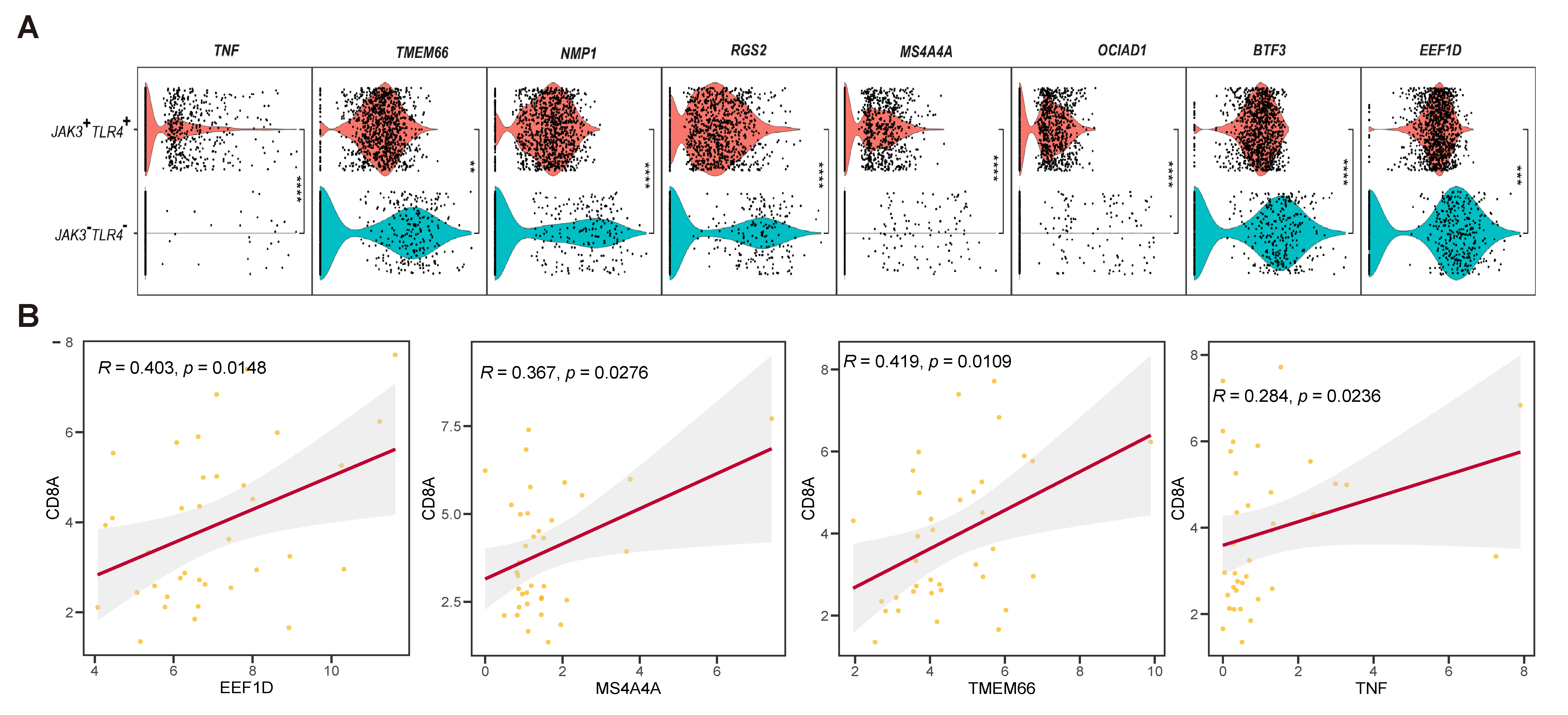

Supplement: Supplementary Figure 9 — JAK3-TLR4- CD16 monocytes are pro-immune. (A) Violin plots showing the expression of JAK3 - TLR4 - mast cluster-specific high expression genes. (B) Scatterplots showing the correlations between the expression of JAK3, TLR4, and CD8A in this study. [file Image_9.tif]

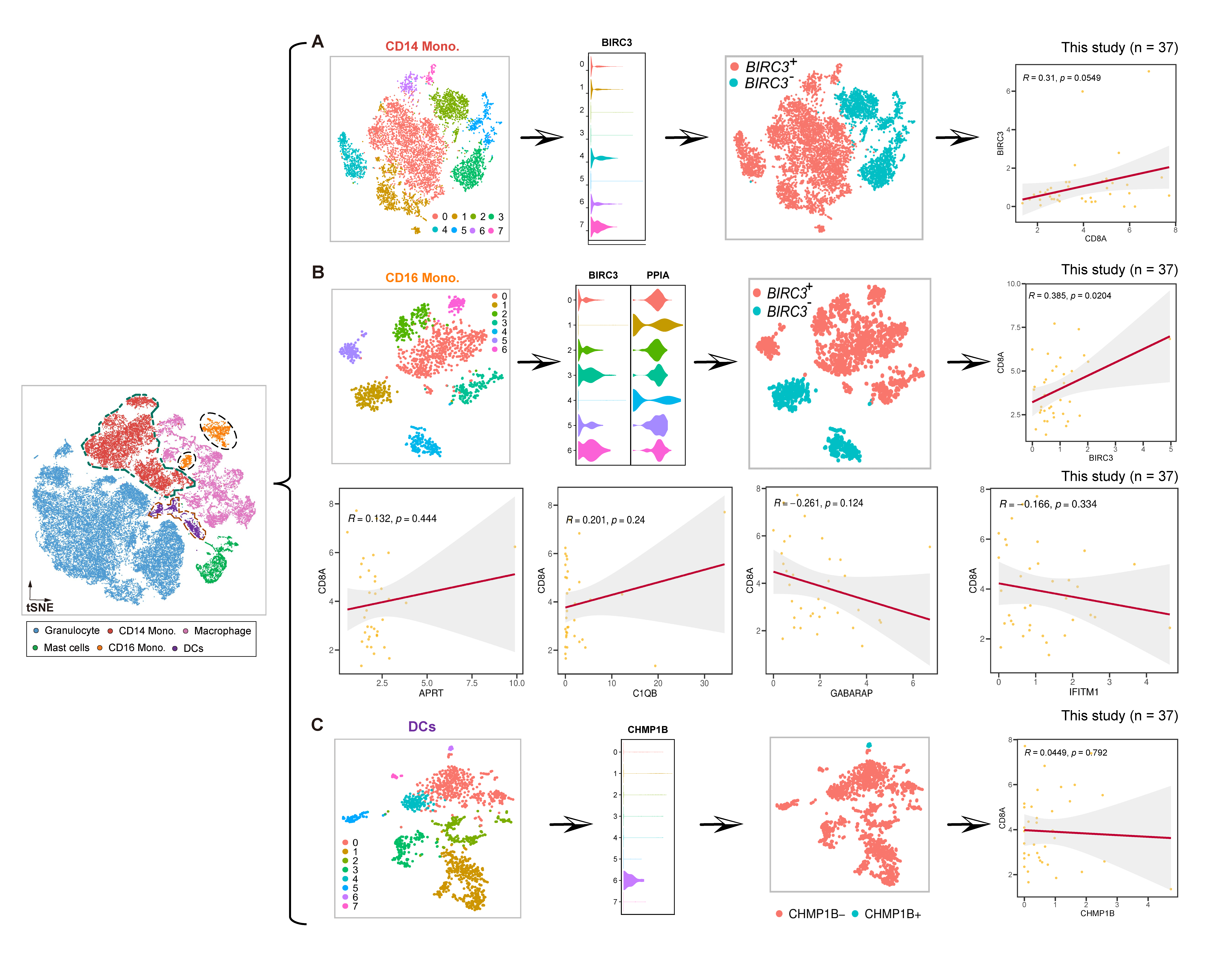

Supplement: Supplementary Figure 10 — Several myeloid subsets may act as pro/anti-immune regulators in a non-necroptosis way. Workflow showing the procedures to distinguish CD14 monocyte (A), CD16 monocyte (B), and DCs (C) by BIRC3, PPIA, and CHMP1B, respectively. [file Image_10.tif]

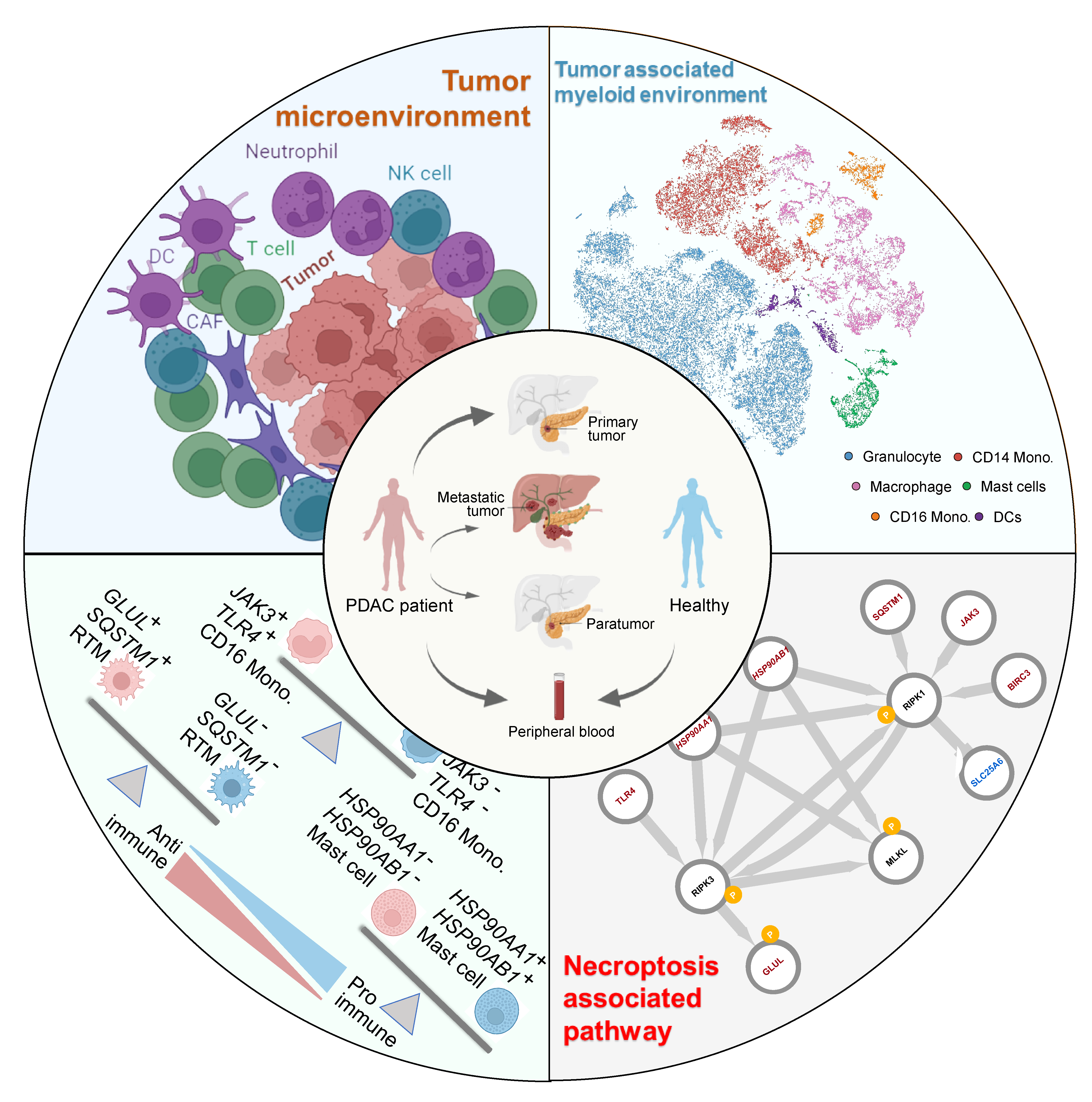

Supplement: Supplementary file 11 [file Image_11.tif]
